# HuRdling Senescence: HuR Breaks BRAF-Induced Senescence in Melanocytes and Supports Melanoma Growth

**DOI:** 10.3390/cancers12051299

**Published:** 2020-05-21

**Authors:** Janika K. Liebig, Silke Kuphal, Anja Katrin Bosserhoff

**Affiliations:** 1 Institute of Biochemistry, Emil-Fischer Zentrum, Friedrich-Alexander University of Erlangen-Nürnberg (FAU), 91054 Erlangen, Germany; Janika.Liebig@fau.de (J.K.L.); silke.kuphal@fau.de (S.K.); 2 Comprehensive Cancer Center (CCC) Erlangen-EMN, 91054 Erlangen, Germany

**Keywords:** malignant melanoma, HuR, oncogene induced senescence, MITF, Microphthalmia-associated transcription factor

## Abstract

In addition to genetic changes, post-transcriptional events strongly contribute to the progression of malignant tumors. The RNA-binding protein HuR (*ELAVL1*) is able to bind and stabilize a large group of target mRNAs, which contain AU-rich elements (ARE) in their 3′-untranslated region. We found HuR to be upregulated in malignant melanoma in vitro and in vivo, significantly correlating with progression in vivo. Additionally, we could show that miR-194-5p can regulate HuR expression level. HuR knockdown in melanoma cells led to the suppression of proliferation and the induction of cellular senescence. Interestingly, HuR overexpression was sufficient to inhibit senescence in *BRAF^V600E^*-expressing melanocytes and to force their growth. Here, MITF (Microphthalmia-associated transcription factor), a key player in suppressing senescence and an ARE containing transcript, is positively regulated by HuR. Our results show for the first time that the overexpression of HuR is an important part of the regulatory pathway in the development of malignant melanoma and functions as a switch to overcome oncogene-induced senescence and to support melanoma formation. These newly defined alterations may provide possibilities for innovative therapeutic approaches.

## 1. Introduction

Until a few decades ago, malignant melanoma was a rather rare tumor disease. There is hardly any other tumor entity, in which incidence rates rise as fast as those for melanoma in recent years [1]. Since the development of immune checkpoint blockade and BRAF inhibition approaches, melanoma therapy has changed [2]. However, even though these approaches show efficacy in many patients, more than half of the patients have to face the consequences of inefficient cancer therapy and therapy resistance [3]. Furthermore, many patients show no response at all. Therefore, a better understanding of the development and progression of melanoma is essential.

An important player in both the development and progression of melanoma is the constitutive activation of MAPK signaling. Approximately 60% of melanomas carry an oncogenic *BRAF* mutation, with *BRAF^V600E^* being the most prevalent [4,5]. Interestingly, not only many melanomas but also approximately 80% of melanocytic nevi harbor such a mutation, however, alone not being sufficient for melanoma development [6,7]. In melanocytes, oncogenic BRAF signaling induces a cell cycle arrest known as oncogene-induced senescence (OIS) [8], which acts as a safeguard mechanism to prevent tumor development. Understanding the mechanisms of development and overcoming senescence will lead to a better knowledge of melanoma formation.

Senescence, a condition found in post-mitotic cells, serves as a protective stress response that limits the replication of aged, damaged, or neoplastic cells [9] via a stable cell cycle arrest in the G_0_/G_1_-phase with simultaneously maintained metabolic activity. By chromosomal condensation, senescent cells commonly form senescence-associated heterochromatic foci (SAHF), which can lead to a stable downregulation of many pro-proliferative genes and thereby to a growth arrest [10]. The senescent phenotype of cells is highly heterogeneous, resulting in the use of multiple markers to analyze senescence.

The dysregulation of post-transcriptional processes is an important factor in the progression of malignant tumors. RNA binding proteins (RBPs) are able to influence every step of transcript processing, including splicing, translation, and change of localization and stability. In doing so, RBPs can both act as stabilizing (e.g., ELAVL proteins) or destabilizing (e.g., AUF1 and TTP) molecules, resulting in the complex regulation of transcripts [11]. An important key element in RBP mode of action are adenine-uridine-rich elements (ARE), commonly found in the 3′ untranslated region (UTR) of mRNAs [12,13]. These elements are defined as regions with a high frequency of adenine and uridine bases. Via these ARE motives, RBPs can fine-tune mRNA stability as a response to extra- and intracellular stimuli. ARE-containing transcripts often occur in short-lived transcripts of early response genes like cytokines, cell cycle regulators, and proto-oncogenes [12].

The ubiquitously expressed ARE-binding protein HuR belongs to the mammalian Hu/ELAV family of RNA binding proteins (RBPs) and was first described in Drosophila as *elav* (embryonic lethal, abnormal vision). The human *HuR/ELAVL1* gene is located on chromosome 19p13.2 and encodes a 32 kDa protein containing the three highly conserved RNA-binding domains RPM-1, RPM-2 and RPM-3. RPM-3 is responsible for binding to the poly(A) tail in the 3′-untranslated region (UTR) of target mRNAs, whereas RPM-1 and RPM-2 bind to AU-rich elements (ARE) in these 3′UTRs. Via this interaction, HuR is known to stabilize target mRNAs [14]. With many targets being mRNAs that encode for proteins important for cell growth, angiogenesis, tumorigenesis, and metastasis, HuR overexpression is known to correlate with poor prognosis in some cancer types [15,16,17]. In malignant melanoma, HuR is discussed as a prognostic marker [18]. However, little is known about the importance of HuR in the development and progression of this cancer type.

In this study, we were able to prove that HuR not only holds a pro-tumorigenic function in melanoma but also bears the capacity to break oncogene-induced senescence in melanocytes via, amongst others, upregulation of MITF and thereby might be involved in the development of melanoma.

## 2. Results

### 2.1. ARE Containing mRNAs Are More Abundant in Melanoma Cells Compared to NHEMs

Initially, we analyzed changes in the mRNA level of transcripts in different melanoma cell lines (primary tumor (PT): Mel Ho, A375; metastasis (Met): 501 Mel, Lu 1205) compared to normal human epidermal melanocytes (NHEMs). We calculated mRNA expression values of 28,536 genes in NHEMs and primary and metastatic melanoma cells based on cDNA array data (GEO: GSE108969) [19]. In comparison to expression values in NHEMs (mean of values set 1), we found more genes to be upregulated than downregulated in melanoma cell lines with the mean values of PT/NHEMs and Met/NHEMs > 2-fold (Figure S1).

**Figure 1:**
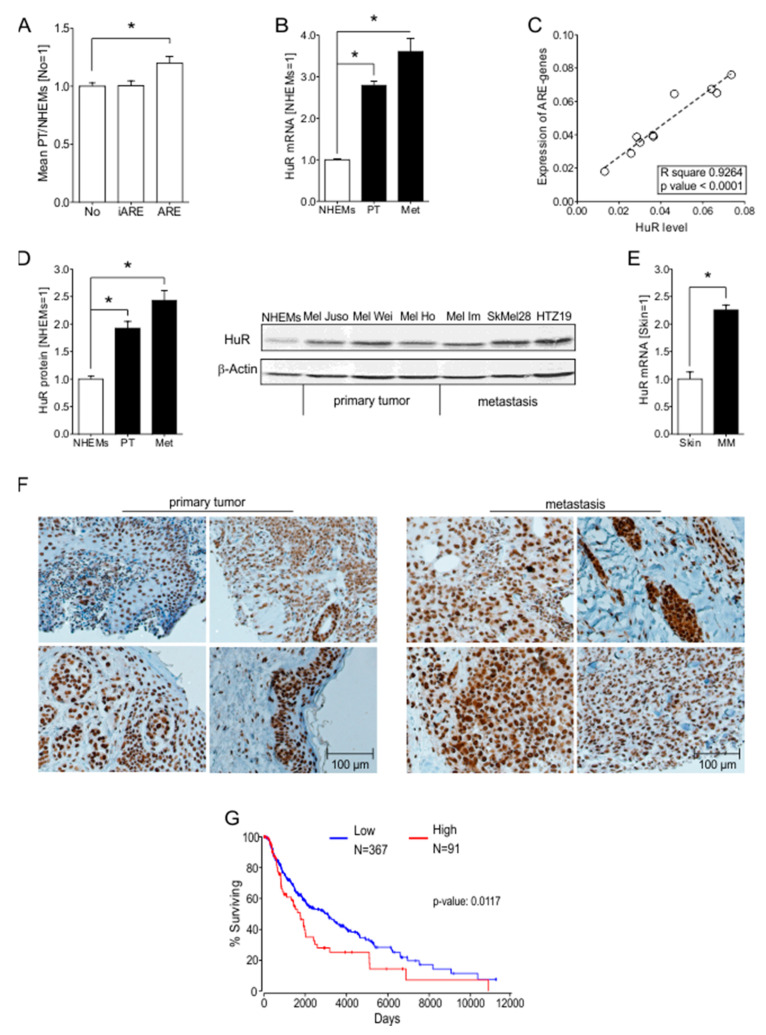
HuR (*ELAVL1*) expression in malignant melanoma in vivo and in vitro. (**A**) Mean expression of mRNAs in primary melanoma cells related to expression in NHEM cells. No = mRNAs without ARE binding sequence (*n* = 9993); iARE = mRNAs with ≥1 intronic ARE sequence (*n* = 5560); ARE = mRNAs with ≥ 1 ARE sequence in the 3′UTR (*n* = 2095). (**B**) Relative expression of HuR mRNA in NHEMs and melanoma cell lines, mRNA level in NHEMs is set 1. (**C**) Correlation of HuR expression, and the mean expression of 150 randomly chosen ARE containing mRNAs in 10 different melanoma cell lines. (**D**) Densitometric quantification (left) and exemplary image (right) of Western blot analysis of HuR protein levels in primary and metastatic melanoma cell lines compared to NHEMs. HuR protein level in NHEMs is set 1. (**E**) Relative expression of HuR mRNA in normal skin (*n* = 7) and melanoma tissue (*n* = 45) of patients. HuR mRNA level in normal skin is set 1. (**F**) Representative immunohistochemical staining of HuR protein in primary human melanoma and metastatic melanoma tissue samples (4 shown, *n* = 10; for quantification see Figure 3). (**G**) Survival analysis in a skin cutaneous melanoma (SKCM) patient dataset comparing low and high HuR (ELAVL1) levels (lower percentile: 80, higher percentile: 20). *A *p*-value of <0.05 was considered statistically significant. The uncropped blots and molecular weight markers of Figure 1D are shown in Figure S5.

In general, apart from elevated transcription, high transcript levels were mainly the result of changed mRNA stability. One of the most common determinants of RNA stability in mammalian cells are AU-rich elements (AREs). An alignment of the cDNA array data with a list of all ARE-containing transcripts (http://brp.kfshrc.edu.sa/ARED/; 3 November 2019) revealed that the number of transcripts containing 3′UTR ARE-sequences was significantly upregulated compared to those without ARE-sequences (Figure 1A). Bearing intronic ARE-sequences did not correlate with the mRNA levels of the corresponding transcripts.

### 2.2. HuR (ELAVL1) Is Upregulated in Malignant Melanoma In Vitro and In Vivo

Focusing on the relevance of ARE containing transcripts, we observed the RNA binding protein (RBP) HuR to be dysregulated in melanoma cells. The primary (Mel Ho and A375) and metastatic melanoma (501 Mel and Lu 1205) cell lines showed a significant upregulation of HuR mRNA compared to NHEMs (Figure 1B) in the cDNA array data. Correspondingly, we were able to prove a significant correlation between the average expression levels of 150 randomly chosen ARE-genes (Table S1) with the HuR mRNA levels in 10 different melanoma cell lines (Mel Ho, A375, 501 Mel, Lu 1205, WM3211, Sbcl2, WM793, WM1366, WM1158, WM9) (Figure 1C). As an RBP that positively regulates ARE-containing transcripts, we chose to investigate HuR and its influence on malignant melanoma further.

To assess HuR protein levels, we screened lysates of NHEMs, primary melanoma (Mel Juso, Mel Wei, and Mel Ho), and metastatic melanoma cell lines (Mel Im, SkMel28, HTZ19). The protein expression was significantly upregulated (Figure 1D) in all melanoma cell lines compared to NHEMs. Interestingly, HuR mRNA and protein expression were most abundant in cell lines derived from melanoma metastases (Figure 1B,D). Supporting the in vitro results, data obtained from GEO profiles showed an in vivo upregulation of HuR mRNA in malignant melanoma patient tissue samples (*n* = 45) compared to normal skin samples (*n* = 7) (data set GDS1375/201727_s_at) (Figure 1E). Besides this data set, additional GEO profiles data sets were found to support this result (GDS1989/201726_at, data not shown). In vivo analysis of HuR protein expression by immunohistochemical staining in primary and metastatic tumor tissue showed HuR expression in all tissue samples, with high expression levels in primary and even higher in metastatic tissues (Figure 1F). By using OncoLnc (http://www.oncolnc.org; 2019/11/03) we analyzed survival data obtained from *The Cancer Genome Atlas* (TCGA) project with expression data from a human skin cutaneous melanoma (SKCM) data set. Here, we were able to show that an elevated level of HuR significantly correlates with lower overall survival of melanoma patients (Figure 1G).

### 2.3. The Loss of miR-194-5p Expression in Melanoma Promotes the Upregulation of HuR

**Figure 2:**
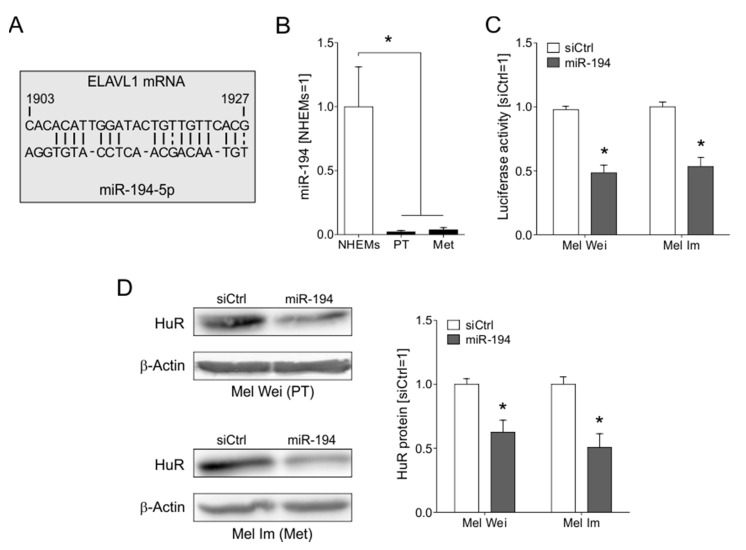
Regulation of HuR (*ELAVL1*) expression by miR-194-5p. (**A**) MiR-194-5p binding site in the 3′UTR of ELAVL1 mRNA. (**B**) MiR-194-5p expression levels in primary and metastatic melanoma cell lines compared to NHEMs. (**C**) Luciferase HuR 3′UTR-reporter activity in siCtrl and miR-194-5p-mimic transfected melanoma cells. Activity in siCtrl transfected cells is set 1. (**D**) Densitometric quantification (**right**) and exemplary image (**left**) of Western blot analysis of HuR protein levels in siCtrl and miR-194-5p-mimic transfected melanoma cells. HuR protein level in siCtrl transfected cells is set 1. *A *p*-value of <0.05 was considered statistically significant. The uncropped blots and molecular weight markers of Figure 2D are shown in Figure S6.

HuR is known to be regulated on post-transcriptional levels [20,21,22,23]. We screened for microRNAs bearing binding sites in the 3′UTR of the HuR mRNA, which was downregulated in malignant melanoma. One miRNA identified in this search was miR-194-5p (Figure 2A), which was almost lost in malignant melanoma cells (PT: Mel Juso, Mel Wei, Mel Ho; Met: Mel Im, SkMel28, HTZ19) compared to NHEMs (Figure 2B). Mimic transfection of this microRNA of melanoma cell lines Mel Wei and Mel Im led to a significant decrease in the activity of a HuR3′UTR luciferase construct (Figure 2C). Accordingly, the overexpression of miR-194-mimic led to reduced HuR protein level (Figure 2D) in both cell lines. 

### 2.4. HuR Expression and Localization Changes in the Progression of MM In Vitro and In Vivo

**Figure 3:**
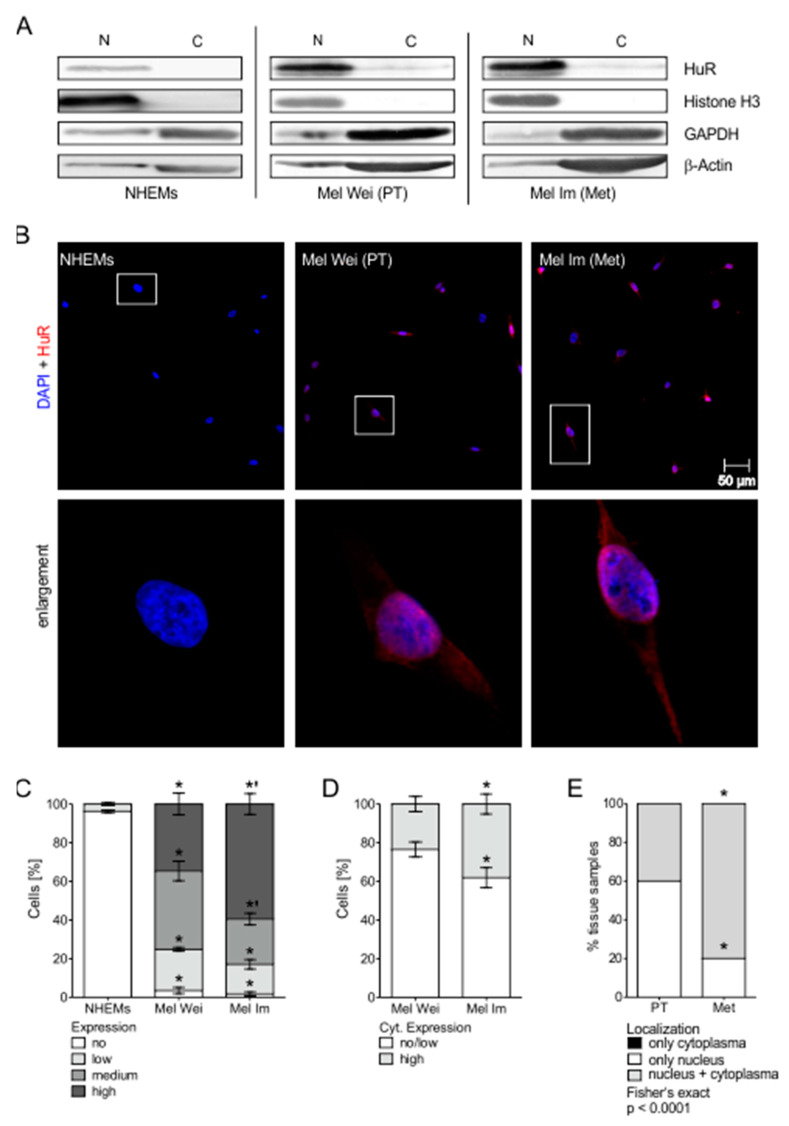
Changes of HuR expression and localization in the progression of MM in vitro. (**A**) Fractionation of cell lysates of NHEM, Mel Wei, and Mel Im cells in nucleus and cytoplasm fraction and subsequent Western blot analysis of HuR protein. Histone H3 served as a nucleus control, GAPDH and β-actin as cytoplasm controls. Representative pictures shown. (**B**) Representative immunofluorescence pictures of NHEM, Mel Wei, and Mel Im cells stained with DAPI (blue) and HuR (red). (**C**) Percentage of no, low, medium, or high HuR level in IF-staining in NHEM, Mel Wei, and Mel Im cells. Significant comparisons with NHEMs were marked with *, significant comparisons between Mel Wei and Mel Im with ‘. (**D**) Percentage of only nuclear/low cytoplasmic and high cytoplasmic HuR level in IF-staining in Mel Wei and Mel Im cells. (**E**) Percentage of only nuclear/nuclear and cytoplasmic HuR localization in IH-staining of primary tumor and metastasis melanoma tissue samples (see Figure 1F). *A *p*-value of <0.05 was considered statistically significant. The uncropped blots and molecular weight markers of Figure 3A are shown in Figure S7.

Further, we analyzed the localization of HuR in melanoma cell lines and in melanocytes. Here, we performed fractionation of cell lysates of NHEM, Mel Wei, and Mel Im cells into nucleus and cytoplasmic fractions. We analyzed these fractions by Western blot analysis and observed strong nuclear localization of HuR in both melanoma cell lines. Additionally, we observed cytoplasmic localization in both melanoma cell lines but not in NHEMs (Figure 3A). By immunofluorescence staining with an anti-HuR antibody very low or no nuclear HuR staining was detected in NHEM. In agreement with data discussed in Figure 1, we observed a higher expression of HuR in Mel Wei compared to NHEM cells. In the metastatic cell line Mel Im, the expression was found to be upregulated even further (Figure 3B,C). Not just the expression but also the localization changed with progression, showing cytoplasmic localization in melanoma cell lines, with Mel Im presenting higher cytoplasmic levels than Mel Wei (Figure 3B,D). In vivo, localization was changed in metastatic compared to primary tumor tissue. In primary melanoma tissue, immunohistochemical staining of HuR was observed in the nuclei of the cells and rarely in the cytoplasm. In metastatic tumor tissue we observed more cytoplasmic staining compared to primary tumors (Figure 3E).

### 2.5. The Knockdown of HuR Leads to Decreased Proliferative Capacity of Melanoma Cells In Vitro

**Figure 4:**
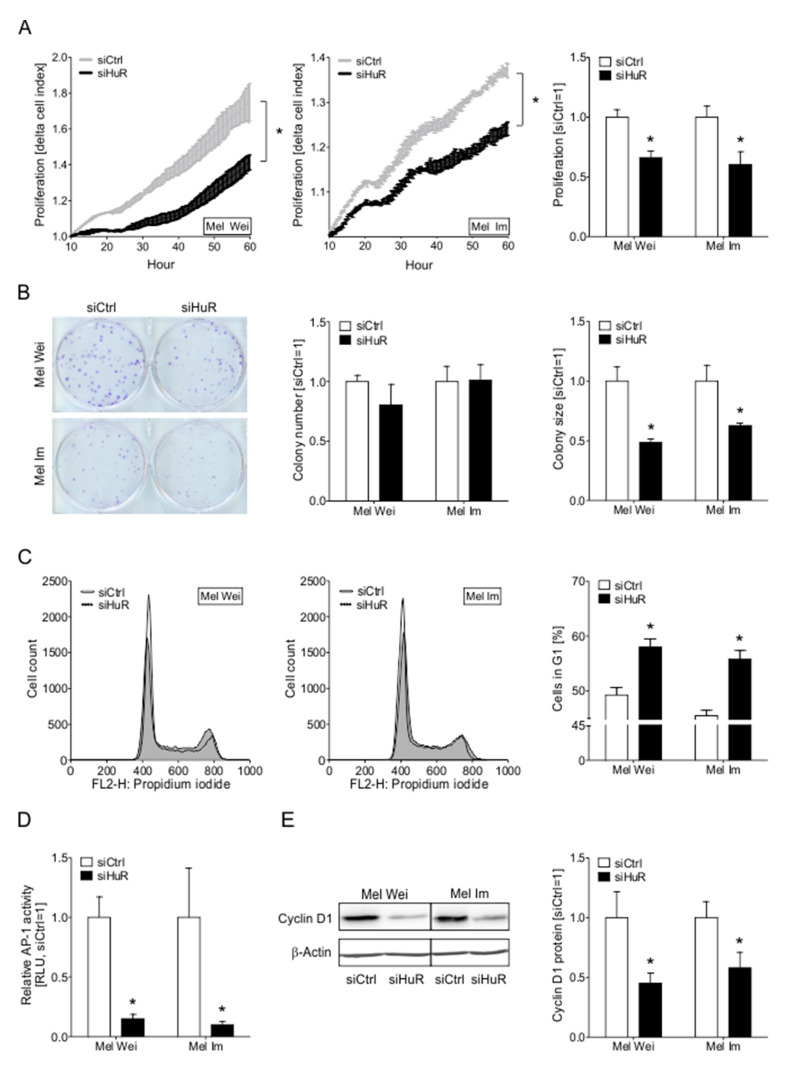
Influence of HuR knockdown on the proliferative capacity of melanoma cells in vitro. (**A**) Exemplary real-time cell proliferation curves of Mel Wei and Mel Im cells (left panel) and quantified ‘slope’ (proliferative ability, siCtrl = 1) (right panel). (**B**) Exemplary images of anchorage-dependent clonogenic assays of Mel Wei and Mel Im cells (left panel) and quantification of colony number and size (right panel) in these cells (siCtrl = 1). (**C**) FACS-based cell cycle analysis of Mel Wei and Mel Im cells. Depicted are representative overlays of cell cycle histograms (left panel) and percentages of G1 cell cycle fractions (right panel) in these cells (siCtrl = 1). (**D**) Luciferase AP-1-reporter activity in siCtrl and siHuR transfected melanoma cells (siCtrl = 1). (**E**) Densitometric quantification (right) and exemplary image (left) of Western blot analysis of Cyclin D1 protein levels in transfected Mel Wei and Mel Im cells (siCtrl = 1). *A *p*-value of <0.05 was considered statistically significant. The uncropped blots and molecular weight markers of Figure 4E are shown in Figure S8.

To further unravel the role of increased HuR expression in melanoma cells, we next analyzed the functional impact of HuR suppression in melanoma cells using a siPool specifically targeting HuR (Figure S2A). The effect of HuR knockdown on the proliferation of Mel Wei and Mel Im melanoma cells was determined using the xCELLigence real-time cell analysis (RTCA) system. HuR knockdown (KD) showed an inhibitory effect on the growth of both cell lines (Figure 4A). With an XTT assay, we were able to confirm these findings (Figure S2B). In a clonogenic assay, the ability of cells to form colonies from single cells was analyzed. After HuR KD, no effect on the clonogenicity of both cell lines was observed, as transfected cells showed the same colony number as the control cells. However, HuR suppression led to smaller colonies in both cell lines (Figure 4B). This finding further emphasizes the effect of HuR knockdown on the proliferation of melanoma cells and shows that the proliferative effects in the preceding assays did not reflect cell death. Additionally, PI/Annexin staining of cells showed no significant changes in apoptosis after siHuR (Figure S2C). We further analyzed the cell cycle by flow cytometry and revealed an increase of siHuR transfected Mel Wei and Mel Im cells in the G_1_/G_0_ phase compared to control cells hinting to a G_1_/G_0_ cell cycle arrest after HuR KD (Figure 4C).

On a molecular level, we observed a reduction in the activity of the pro-proliferative transcription factor AP-1 (Figure 4D). Additionally, we were able to show that Cyclin D1 protein, a factor important for G_1_/S transition, is downregulated in siHuR transfected melanoma cells (Figure 4E). These data indicated that loss of HuR results in reduced cell proliferation by induction of a G_1_/G_0_ cell cycle arrest.

### 2.6. HuR Knockdown Induces Senescence Characteristics in Human Melanoma Cells

**Figure 5:**
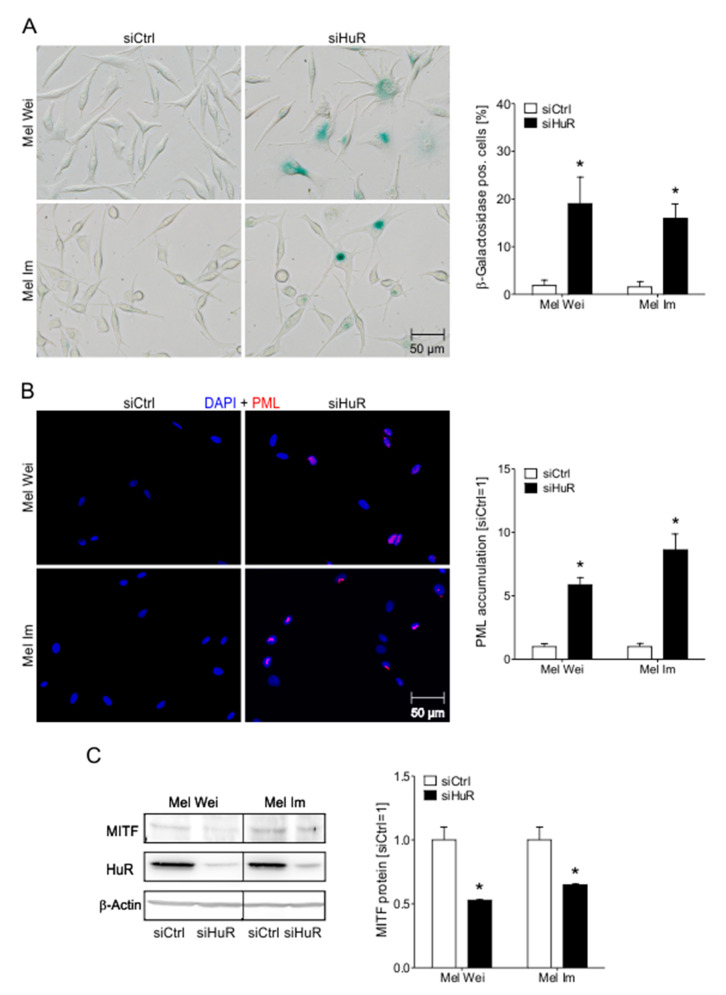
Influence of a HuR knockdown on senescence characteristics in melanoma cells. (**A**) Exemplary images of light microscopic examination of SA-β-galactosidase staining in Mel Wei and Mel Im cells (left). The percentages of SA-β-galactosidase positive cells (blue) were calculated (right) (siCtrl = 1). (**B**) Exemplary images of PML immunofluorescence staining of Mel Wei and Mel Im cell lines. Panels show overlays of PML (red) and DAPI (blue) staining (left). The graph shows the nuclear accumulation of PML (right) (siCtrl = 1). (**C**) Densitometric quantification (right) and exemplary image (left) of Western blot analysis of MITF protein levels in transfected Mel Wei and Mel Im cells (siCtrl = 1). *A *p*-value of <0.05 was considered statistically significant. The uncropped blots and molecular weight markers of Figure 5C are shown in Figure S9.

Strikingly, after knockdown of HuR, the melanoma cell lines developed morphological alterations reminiscent of senescent cells, such as enlarged cell bodies and partially branched cellular extensions (Figure S3A). To investigate whether the cell cycle arrest of Mel Wei and Mel Im melanoma cells after the suppression of HuR is based on induction of senescence, we performed senescence-associated (SA)-β-galactosidase staining. Interestingly, the SA-β-galactosidase staining was strongly induced after HuR knockdown in both cell lines compared to control cells (Figure 5A), suggesting a strong formation of a senescence-like phenotype under HuR suppression. Since it is well known that etoposide induces premature senescence [24], etoposide was used as a positive control (Figure S3B). Senescent cells often show the formation of so-called senescence-associated heterochromatin foci (SAHF). These SAHF are formed by chromatin restructuring and commonly lead to the stable downregulation of pro-proliferative genes and to cell cycle arrest. The promyelocytic leukemia protein (PML), a protein functionally implicated in cellular senescence in melanoma, is involved in the development of these SAHF [25,26]. SiHuR-transfected melanoma cells showed a strong increase in PML accumulation (Figure 5B). Histon-deacetylases and –methyltransferases are also involved in the chromosomal condensation and thereby formation of SAHF. Trimethylated histone 3 can be used as a marker for hypermethylation [10,27]. Immunofluorescence staining showed a significant upregulation of tri-methylated H3K9 in HuR suppressed melanoma cells (Figure S3C), further proving an induction of senescence.

The induction of senescence by HuR suppression was in line with an MITF^low^ phenotype in these cells (Figure 5C). MITF, known to negatively impact the program of senescence itself, contains ARE sequences in its 3′UTR, suggesting an effect of HuR in MITF mRNA stabilization.

### 2.7. HuR Overexpression in NHEM BRAF^V600E^ Cells Is Capable to Bypass Oncogene Induced Senescence by Induction of an MITF^high^ Phenotype

**Figure 6:**
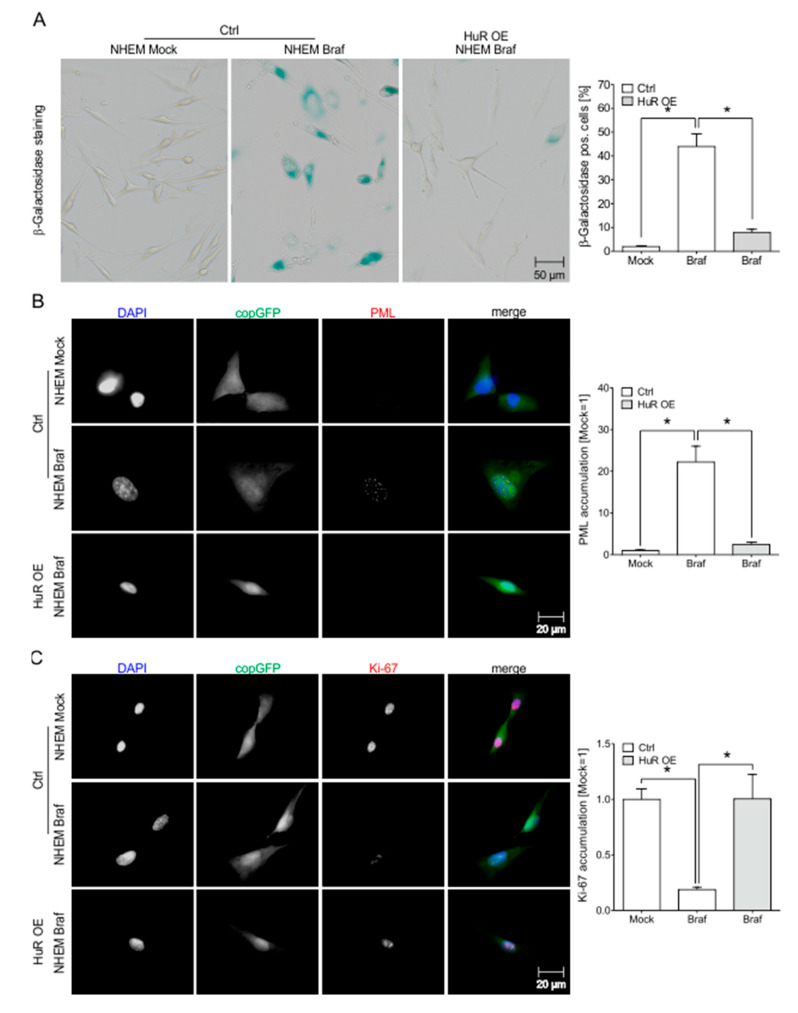
HuR overexpression leads to a reduction of senescence characteristics in NHEM *Braf^V600E^* cells. (**A**) Exemplary images of light microscopic examination of SA-β-galactosidase staining in NHEM Mock, NHEM *Braf^V600E^*, and NHEM *Braf^V600E^*/HuR OE cells (left). The percentages of SA-β-galactosidase positive cells (blue) were calculated (right) (Mock = 1). (**B**) Exemplary images of PML immunofluorescence staining of NHEM Mock, NHEM *Braf^V600E^*, and NHEM *Braf^V600E^*/HuR OE cells (red) and DAPI (blue) staining. CopGFP served as a transduction control (green) (left). The graph shows the nuclear accumulation of PML (Mock = 1) (right). For better recognition, colors are only shown in the overlay, not in single pictures. (**C** Exemplary images of Ki-67 immunofluorescence staining of NHEM Mock, NHEM *Braf^V600E^*, and NHEM *Braf^V600E^*/HuR OE cells (red) and DAPI (blue) staining. CopGFP served as a transduction control (green) (top). The graph shows the accumulation of Ki-67 (Mock = 1) (bottom left). For better recognition, colors are only shown in the overlay, not in single pictures. *A *p*-value of <0.05 was considered statistically significant.

The loss of HuR appears to be a driving force for senescence induction in malignant melanoma. To understand whether the induction of HuR expression alone is able to overcome senescence in HuR^low^ cells, we analyzed overexpression of HuR in the oncogene-induced senescence (OIS) model NHEM *BRAF^V600E^* [8] by lentiviral overexpression (OE) of HuR in NHEM *BRAF^V600E^* and NHEM Mock cells (Figure S4A,B). Lentiviral overexpression of HuR led to reduced SA-β-galactosidase staining in NHEM *BRAF^V600E^* cells (Figure 6A). Additionally, increased HuR expression was capable of reversing the induction of PML (Figure 6B) and tri-methylated H3K9 (Figure S4D) in NHEM *BRAF^V600E^* cells. By immunofluorescence staining of the proliferation marker Ki-67 (Figure 6C) and by assessing the cell numbers 1 week after transduction of the cells (Figure S4D), we were able to show that HuR overexpression is capable of reversing the anti-proliferative effect of *BRAF^V600E^* OIS in NHEM cells.

**Figure 7:**
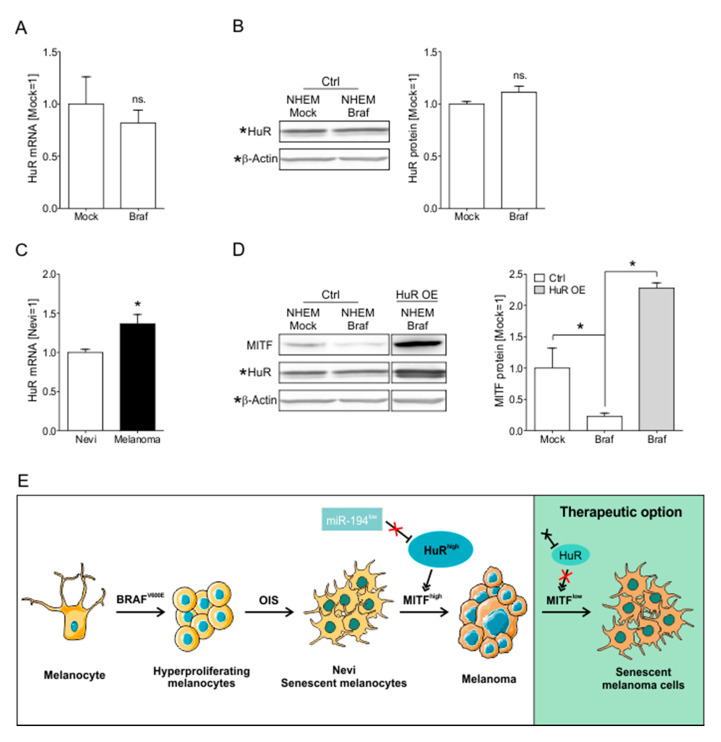
HuR overexpression leads to MITF^high^ phenotype NHEM *Braf^V600E^* cells. (**A**) Relative expression of HuR mRNA in NHEM Mock and NHEM *Braf^V600E^* cells (Mock = 1). (**B**) Densitometric quantification (left) and exemplary image (right) of Western blot analysis of HuR protein levels in NHEM Mock and NHEM *Braf^V600E^* cells (Mock = 1). (**C**) Relative expression of HuR mRNA in matched Nevi and melanoma tissue samples (Nevi = 1). Data derived from the GSE112509 data set. (**D**) Exemplary image of Western blot analysis of MITF protein in NHEM Mock, NHEM *Braf^V600E^*, and NHEM *Braf^V600E^*/HuR OE cells (left). Densitometric quantification of MITF protein in NHEM Mock, NHEM *Braf^V600E^*, and NHEM *Braf^V600E^*/HuR OE cells (Mock = 1) (right) (B&D represent the same Western blot, which was duplicated for presentation purposes, * marks lanes already presented in B). (**E**) Schematic summary of the main results (schematic illustrations abstracted and modified from https://smart.servier.com/, for licensing, please refer to the material and method part of this work). *A *p*-value of <0.05 was considered statistically significant. The uncropped blots and molecular weight markers of Figure 7D are shown in Figure S10.

Interestingly, we could not detect any changes in the HuR mRNA (Figure 7A) or protein (Figure 7B) level in NHEM *BRAF^V600E^* compared to NHEM Mock cells, proving that HuR is not involved in OIS in NHEM.

By overcoming OIS, melanoma can develop from melanocytic nevi. Interestingly, in RNA-seq analysis of nevi (*n* = 23) and melanoma (derived from nevi, *n* = 57) tissue samples [28] we found HuR to be significantly upregulated in nevi-derived melanomas compared to nevi, further suggesting an effect of HuR induction in overcoming of OIS and melanoma induction (Figure 7C). By Western blot analysis, we could show that HuR OE led to an upregulation of MITF mRNA and thereby to a MITF^high^ phenotype in NHEM *BRAF^V600E^* cells (Figure 7D).

In line with these data, we propose that HuR functions as a potential stabilizer of MITF, thereby influencing the proliferation and the senescent phenotype of cells. With this capacity, HuR is sufficient to bypass or potentially overcome OIS in melanocytes and could thereby be involved in the development of nevi-derived melanoma (Schematic summary Figure 7E).

## 3. Discussion

The post-transcriptional regulation of mRNA by RNA binding proteins (RBP) is a fast and effective way to adapt the proteome of a cell to changing external stimuli and is an important factor in the development of cancers [29,30]. Changes in the expression of these RBPs are known to be involved in the development and progression of different cancer types, such as breast, lung, ovarian, and colon cancer [31,32]. An important group of RBPs, ARE-binding proteins, bind to AU-rich elements in target mRNAs. As a general phenomenon in melanoma, we found many stabilized mRNAs harboring ARE-sequences. Usually, ARE-elements lead to rapid mRNA degradation. However, the expression of HuR, a stabilizing ARE BP, was revealed to be induced in melanoma in vivo and in vitro, thereby potentially stabilizing the targeted mRNAs. HuR transcript levels correlated with the number of transcripts in melanoma cells, additionally indicating an important role of HuR in the induction of ARE containing mRNAs. 

Little is known about the abundance and impact of HuR in melanoma cells. It was shown before that HuR is expressed in melanoma cell lines and that it is localized both in the nucleus and cytoplasm of these cells [33]. However, this study was conducted without referring to melanocytes as ‘healthy’ melanoma precursor cells, therefore, not stating deregulation. Our data showed that in melanocytes, HuR expression was absent or very low, and if expressed, mainly nucleic. In the melanoma cell lines, we observed an upregulation of protein expression and a shift to the cytoplasm. In brain tumor and non-small cell lung carcinoma cells, HuB (ELAVL2), the only neural ELAVL protein found to be expressed in cancer cells, was shown to initiate the cytoplasmic translocation of HuR. Afterward, HuB and HuR form a complex in the cytoplasm to enable the stabilization of ARE transcripts by HuR [34]. HuB mRNA is upregulated in primary and metastatic melanoma cell lines compared to NHEMs (GEO: GSE108969) [19]. This opens the possibility that HuB is responsible for the elevated cytoplasmic expression of HuR, and the changed ARE mRNA stability that we found in the analyzed melanoma cell lines.

Research on other diseases suggests that the localization of HuR is of major importance for the function of this RBP. In studies on cobalamin metabolism disorders, the nucleocytoplasmic transport of HuR was shown to be disrupted as a result of dephosphorylation and hypomethylation of HuR, leading to decreased expression of genes involved in the cellular stress response, cell cycle regulation, neurogenesis, and neuroplasticity [35]. In a zebrafish model, changed phosphorylation of HuR led to the disruption of translocation and thereby to less stable *gata1* mRNA and impaired formation of red blood cells [36]. Interestingly, high cytoplasmic localization of HuR is known to correlate with bad prognosis in several cancer types, including breast and lung [15,37] and now also in melanoma. The enhanced cytoplasmic localization of HuR in metastasis compared to the primary tumor was not discussed in the literature before—it might further promote tumor progression by prolonging mRNA stability.

The dysregulation of RBPs is often the result of epigenetic events or microRNA-dependent regulation [20,38]. Our in silico analyses identified a miR-194 binding site in the 3′UTR of the HuR transcript, and miR-194 was proven to function as a tumor suppressor microRNA, found to be almost lost in melanoma cell lines. Overexpression of a miR-194-specific mimic directly repressed HuR expression in the melanoma cell lines proving a regulatory function. Some microRNAs are already known to influence HuR in different cancer types. MiR-519, miR-125a, and miR-16 are known to repress HuR protein expression in a direct manner [20,22,39]. The miR-29a regulates HuR indirectly by the degradation of tristetraprolin (TTP) mRNA, an RBP negatively influencing HuR expression [23]. In melanoma, miR-29a expression is induced [40], whereas miR-125a and miR-16 get lost during melanoma development [41,42]. This leads to the assumption that these miRNAs could be additionally involved in the regulation of HuR in melanoma. There is no study about the regulation and function of miR-519 in melanoma, yet.

Our functional analysis showed that suppression of endogenous HuR in melanoma cell lines inhibited cell proliferation by inducing a G1 cell cycle arrest. In line, we identified the transcription factor AP-1 and its target Cyclin D1, both shown to be affected by HuR in other cancer types, as targets of HuR regulation in melanoma cells. Previously, it has been shown that HuR stabilizes WNT5A and thereby positively influences melanoma cell migration [33]. Additionally, COX-2 mRNA was described as a target of HuR stabilization, leading to a HuR dependent inhibition of apoptosis in melanoma cell lines [43]. In other cancer types, HuR is characterized as a stabilizer of cyclin mRNAs, thereby promoting the proliferation of cancer cells [44,45]. In cervical cancer cells, the mRNA stability of Cyclin D1 is increased by HuR overexpression [46]. In breast cancer, AP-1 expression is induced by increased c-Jun mRNA stabilization as a result of HuR overexpression [47]. In some cases, HuR overexpression in cancer cells seems to be beneficial for therapeutic approaches. Just recently, it was demonstrated that drug-induced overexpression of HuR prevents the development of resistance to BRAFi therapy in melanoma [48].

Detailed analyses of the G1 cell cycle arrest led us to focus on the mechanism of cellular senescence. In the melanoma cell lines used in this study, knockdown of HuR led to the induction of senescence with a change of cell morphology and the induction of several senescence markers. HuR was previously shown to get lost during replicative senescence in human fibroblasts, resulting in a downregulation of many proliferative genes [45]. Further, the loss of HuR expression during replicative senescence was shown to enhance TIN2 protein expression and thereby the production of reactive oxygen species (ROS), which further induce senescence [49]. 

This study shows for the first time that MITF, a master regulator in melanocyte differentiation and pigmentation, is regulated by HuR. Melanoma cells with high cellular MITF-level are proliferative, those with low levels show a stem cell-like invasive phenotype [50]. In addition, the long-term reduction of MITF in melanoma cells leads to the induction of cellular senescence [51,52]. HuR knockdown in the melanoma cell lines led to the suppression of MITF levels and to the induction of senescence. It is not yet known whether MITF is regulated by HuR in a direct or indirect matter, however, it is known that MITF bears an ARE sequence in its mRNA.

In all functional analyses, there was no significant difference detectable between the primary melanoma cell line Mel Wei and the metastasis melanoma cell line Mel Im, leading to the assumption that HuR does not change its function during progression. Induced expression in metastasis may further enhance the expression of proliferative genes and accelerate proliferation. 

The impact of HuR during the development of melanoma might be most important. For nevi-derived melanomas, the hurdling of senescence is a crucial step in cancer development. To address the question of whether HuR not just inhibits the formation of senescence in melanoma but could also bypass a senescent phenotype in nevi, this study investigated for the first time the influence of HuR on oncogene-induced senescence (OIS) in melanocytes. In our study, we observed that the lentiviral induction of HuR in NHEM *BRAF^V600E^* enhanced the proliferative capacity and lead to a revision of the senescent state of NHEM. Additionally, HuR overexpression in senescent melanocytes led to the induction of MITF expression and to a more proliferative state. Additionally, we were able to show a significant upregulation of HuR in nevi-derived melanomas, compared to healthy nevi tissue. Melanocytic nevi arise after oncogene activation by hyperproliferation of melanocytes, where the initial strong proliferation is stopped by the induction of senescence [8,53]. Revision of this senescent state by additional mutations can lead to the formation of malignant melanoma. The capacity of HuR to negatively influence OIS in senescent melanocytes and the upregulated expression of HuR in nevi-derived melanomas suggests HuR to be an important player in the development of malignant melanoma from benign nevi. In normal fibroblasts, the downregulation of HuR during DNA damage-associated senescence has been shown to lead to inhibition of the heat shock transcription factor Hsf1 via downregulation of SIRT1. The loss of Hsf1 downregulates the heat shock response of cells, leaves cells unprotected against proteotoxic stresses, and thereby contributes to the maintenance of cellular senescence [54]. Wang et al. were able to show that HuR is involved in the regulation of several genes whose expression decreases during fibroblast senescence. Overexpression of HuR in senescent fibroblasts restored a “younger” phenotype in the cells, while the reduction of HuR expression further supported the senescent phenotype [45].

In summary, this study shows that a high expression of HuR in melanoma leads to higher proliferation and inhibition of senescence. In *BRAF^V600E^*-transduced melanocytes, HuR overexpression leads to the loss of senescence characteristics and a proliferative phenotype. The influence of HuR on the induction of proliferation in senescent melanocytes suggests an important impact of HuR on the development of melanoma from melanocytic nevi. MITF, regulated by HuR, was found to be a player in HuR-mediated inhibition of senescence. 

Investigation of the molecular mechanisms of HuR regulation could help to identify new targets for melanoma therapy. Currently, inhibition of cytoplasmic accumulation of HuR or disruption of the interaction between HuR and its targets by small molecule inhibitors have been evaluated [55,56]. In colon carcinoma cells, the inhibitor MS-444 was shown to inhibit the tumorigenic potential of HuR, without exerting a considerable effect on healthy intestinal cells [57,58]. Since the translocation of HuR into the cytoplasm is a crucial factor in the pro-tumorigenic activity of HuR, the use of inhibitors like MS-444 could offer a promising approach in melanoma therapy. However, the fact that ARE mRNAs can also be regulated by other RBPs and microRNAs makes it difficult to understand the regulation of ARE-containing transcripts and their contributions to cancer development, progression, and therapeutic response. A thorough understanding of the interplay between RBPs, microRNAs, and their targets could be the key in the development of new therapeutic approaches for the treatment of melanoma.

Concluding, our findings indicate that the changed expression and subcellular localization of HuR in melanoma is of particular importance for the enhanced proliferation and aggressiveness of melanoma cells. Much more importantly, we found HuR to be a potential key player in the hurdling of senescence and thereby the development of malignant melanoma.

## 4. Material and Methods

### 4.1. Cell Lines and Cell Culture Conditions

The human melanoma cell lines Mel Wei, Mel Ho (derived from primary cutaneous melanoma), Mel Im, SkMel28, and HTZ19 (isolated from metastases of malignant melanoma) were maintained in Dulbecco’s modified Eagle’s medium (DMEM) supplemented with penicillin (400 U/mL), streptomycin (50 µg/mL), and 10% fetal calf serum (all from Sigma-Aldrich, München, Germany). For the primary melanoma cell line Mel Juso, RPMI-1640 (Roswell Park Memorial Institute) medium with NaHCO_3_ with the same supplements was used. Normal human epidermal melanocytes (NHEMs) were cultivated in MGM-4 BulletKit medium (Lonza, Basel, Switzerland). All cell lines were incubated at 37 °C in a humidified atmosphere containing 8% (melanoma cell lines) or 5% (NHEMs) CO_2_. The cultivation of all cell lines was described previously [59].

### 4.2. Lentiviral Transduction

Lentiviral transduction was performed as described previously [60]. BRAF^V600E^ and HuR overexpression (OE) plasmids were used, transductions were performed simultaneously. As a control the copGFP plasmid (Mock) was used. For the preparation of the HuR OE plasmid (pCDH-CMV-HuR-EF1-copGFP) we used a GFP-HuR plasmid as the HuR sequence template and the lentiviral pCDH-CMV-MCS-EF1-copGFP (copGFP) vector as the target vector. The primers 5′-CAGGAATTCAAGTCTAATGGTTATGAAGACC-3′ (HuR_w/oGFP_Fwd) and 5′-CAGGCGGCCGCTTATTTGTGGGACTTGTTGGTT-3′ (HuR_w/oGFP_Rev) were used to amplify the HuR sequence, add correct restriction sites, and rebuild the start codon. GFP-HuR was a gift from Christine Mayr (Addgene plasmid #121162; http://n2t.net/addgene:121162; RRID:Addgene_121162) [61].

### 4.3. siRNA Transfection

The melanoma cell lines Mel Wei and Mel Im were transfected with a siPool (about 30 siRNAs) against HuR (functionally verified by siTOOLs Biotech, Planegg/Martinsried, Germany [62]) or a negative control siPool using Lipofectamine RNAiMAX reagent (Life Technologies, Darmstadt, Germany) as described previously [19]. For transfection, 150,000 cells were seeded into the wells of a 6-well plate. Cells were incubated for 72 h.

### 4.4. miRNA Mimic Transfection

The melanoma cell lines Mel Wei and Mel Im were transfected with a microRNA-194-5p mimic (Qiagen, Hilden, Germany) using Lipofectamine RNAiMAX reagent (Life Technologies) as described previously [19]. For transfection, 150,000 cells were seeded into the wells of a 6-well plate. Cells were incubated for 72 h. Allstars Negative Control siRNA (Qiagen) was used as a negative control.

### 4.5. mRNA + miRNA Expression Analysis

Isolation of total cellular RNA from cultured cells and generation of cDNAs by reverse transcription (RT) reaction was performed as described previously [63]. Quantitative real-time PCR (qRT-PCR) analysis of gene expression was performed on a LightCycler 480 system with specific sets of primers (β-Aktin: 5’-CTACGTCGCCCTGGACTTCGAGC-3′, 5′-GATGGAGCCGCCGATCCACACGG-3′; HuR: 5′-TAAGGTGTCGTATGCTCGCC-3′, 5′-CGGATAAACGCAACCCCTCT-3′) as described previously [64].

Isolation of total cellular miRNA from cultured cells and generation of micDNAs by reverse transcription (RT) reaction was performed as described previously [65]. For expression analysis of microRNA-194, qRT-PCR was performed as described previously [66] using miScript microRNA-194 (or control U6) and universal primer (Qiagen, Hilden, Germany).

### 4.6. Subcellular Fractionation

For separation of nuclear and cytoplasmic cell fractions using centrifugation methods, the subcellular fractionation protocol of Abcam (Cambridge, England) was used. Histone H3 was used as a nuclear control, GAPDH and β-actin were used as cytoplasmic controls (List of primary antibodies: Table S2).

### 4.7. Western Blotting

Preparation of complete cell lysates and Western blot analysis was performed as described previously [67]. In brief, 10–30 µg of RIPA (Roche Diagnostics, Penzberg, Germany) was loaded per lane on a 12.75% gel and separated by SDS-PAGE (Table S2: primary antibodies; Table S3: secondary antibodies).

### 4.8. Immunohistochemical Analysis

Standard 5 µm sections of formalin-fixed and paraffin-embedded tissue blocks were used for immunohistochemical analysis of human tissue samples (tissue microarray (TMA) comprising specimens from benign nevi, primary melanoma and melanoma metastases) [64]. Immunohistochemical staining was performed using an anti-HuR antibody (sc-5261, Santa Cruz, TX, USA; 1:50).

Sampling and handling of patient material were carried out in accordance with the ethical principles of the Declaration of Helsinki. The use of human tissue material had been approved by the local ethics committee of the University of Regensburg (application numbers 09/11 and 03/151).

### 4.9. Immunofluorescence Staining

Immunofluorescence analyses of cells were performed as described previously [60] (Table S2: primary antibodies; Table S3: secondary antibodies).

### 4.10. Analysis of Cell Proliferation

Real-time cell proliferation was measured using the xCELLigence System (Roche, Mannheim, Germany) (“E-Plates”) as described earlier [68]. Cell cycle analysis was performed using fluorescence-activated cell sorting (FACS) as described [69]. Moreover, the XTT assay was used as previously described [70].

### 4.11. Clonogenic Assay

Attachment-dependent colony formation and growth of cancer cells were analyzed using clonogenic assays as described before [71]. Both number and size of colonies were determined using the CellSens software (Olympus, Tokio, Japan).

### 4.12. Analysis of Apoptosis by Flow Cytometry

Apoptotic cells were investigated by flow cytometry using the Apoptotic/Necrotic Cells Detection Kit (PromoCell, Heidelberg, Germany) according to the manufacturer’s instructions and as described previously [67]. The flow cytometry analysis was performed in a FACS CALIBUR cytometer (Becton Dickinson Bioscience, Heidelberg, Germany). Flow cytometry data were analyzed using CellQuest Pro Software (BD Bioscience).

### 4.13. Analysis of Intracellular ROS Content by Flow Cytometry

For analysis of ROS content, cells were washed with PBS and subsequently treated with 1 mL 2′,7′-Dichlorofluorescin diacetate (10 µM, H_2_DCFDA) (Sigma-Aldrich, Taufkirchen, Germany) for 15 min (37 °C, 8% CO_2_). After incubation, cells were washed with PBS, and cell pellets were collected. Cells were suspended in PBS, and the flow cytometry analysis was performed in a FACS CALIBUR cytometer (BD Bioscience) using the FL-1 channel. Flow cytometry data were analyzed using CellQuest Pro Software (BD Bioscience).

### 4.14. Luciferase Assay

Cells were seeded in duplicates into 6-well plates and transfected, as mentioned earlier. After 48 h, cells were transiently transfected with 0.5 µg of plasmid DNA (pSGG-empty-3′UTR, pSGG-HuRFL-3′UTR, pGL2-basic, AP-1-LUC) using Lipofectamine Plus (Gibco, Karlsruhe, Germany) according to the manufacturer’s instructions. The cells were lysed 24 h after transfection, and the luciferase activity was quantified by a luminometric assay (Promega Corp., Madison, WI, USA). To determine transfection efficiency, the data were normalized to renilla luciferase activity. Therefore, cells were co-transfected with 0.1 µg of the pRL-TK plasmid (Promega). The vectors pSGG-empty-3′UTR and pSGG-HuRFL-3′UTR were kindly provided by Faoud Ishmael, MD [72].

### 4.15. Senescence-Associated ß-Galactosidase Staining

For senescence-associated ß-galactosidase staining, the Senescence ß-galactosidase Staining Kit (Cell Signaling Technology, Danvers, MA, USA) was used in a blinded fashion as described before [67]. Cells were stained in 6-well culture plates for 24 h (melanoma cells) or 7 h (NHEMs).

### 4.16. Schematic Illustrations

Schematic illustrations were abstracted from “Les Laboratoires Servier—smart” Medical Art (https://smart.servier.com/) and modified. Licensing took place according to the terms of the provider: https://creativecommons.org/licenses/by/3.0/legalcode.

### 4.17. Statistical Analysis

Statistical analysis was performed using GraphPad Prism software package (GraphPad Software Inc., San Diego, CA, USA). Results were expressed as the mean ± SEM or a percentage value. Comparisons between groups were conducted using Student’s unpaired *t*-test. A *p*-value of <0.05 was considered statistically significant (depicted in figures as ‘*’; ns: not significant). If not depicted otherwise in figure legends, the number of independent experiments was *n* ≥ 3.

### 4.18. Patients’ Consent

Sampling and handling of patient material were carried out in accordance with the ethical principles of the Declaration of Helsinki. Use of human tissue material had been approved by the local ethics committee of the University of Regensburg (application numbers 09/11 and 03/151). For the use of patient material without any further information with regards to the patient except for the kind of tumor, no written individual consent is necessary in addition to the approval of the ethics committee. *HuR mRNA Level in melanoma patient tissue samples*: Data obtained from publicly available sources (GEO profiles). *Survival data from skin cancer patients*: Data obtained from publicly available sources (Cancer Genome Atlas, OncoLnc, http://www.oncolnc.org [73]).

## 5. Conclusions

Our results show for the first time that the overexpression of HuR is an important part of the regulatory pathway in the development of malignant melanoma and functions as a switch to overcome or bypass oncogene-induced senescence and to support melanoma formation. These newly defined alterations may provide possibilities for innovative therapeutic approaches.

## Supplementary Materials

The following are available online at https://www.mdpi.com/2072-6694/12/5/1299/s1, Figure S1: Mean expression of mRNAs in NHEMs and primary and metastatic melanoma cells. Data from cDNA array (Gene Omnibus (GEO) database (GSE108969)). Expression in NHEMs = 1. Expression levels of 28536 genes were involved in the calculation, Figure S2: A Transfection control for siHuRpool and siCtrlpool transfection. Relative expression of HuR mRNA in transfected Mel Wei and Mel Im cells (siCtrl = 1) (left). Densitometric quantification (right) and exemplary image (middle) of western blot analysis of HuR protein levels in transfected Mel Wei and Mel Im cells (siCtrl = 1). B Exemplary proliferation curves of Mel Wei and Mel Im cells resulting from a XTT assay (left panel) and quantified ‘OD’ (proliferative ability, siCtrl = 1) (right panel). C FACS-based analysis of apoptosis in transfected Mel Wei and Mel Im cells (siCtrl = 1), Figure S3: A Exemplary images of light microscopic examination of siCtrl and siHuR transfected Mel Wei and Mel Im cells. Enlarged cells marked with asterisks. B Exemplary images of light microscopic examination of SA-β-galactosidase staining in etoposide treated Mel Wei and Mel Im cells (positive control) (right). The percentages of SA β galactosidase positive cells (blue) were calculated (left) (siCtrl = 1). C Exemplary images of H3K9-trimethyl immunofluorescence staining of Mel Wei and Mel Im cells. Panels show overlays of H3K9-trimethyl (red) and DAPI (blue) staining (left). The graph shows the amount of H3K9-trimethyl protein (right) (siCtrl = 1), Figure S4: A Transduction control for HuR OE. Relative expression of HuR mRNA in NHEM Mock, NHEM BrafV600E and NHEM BrafV600E/HuR OE cells (Mock = 1). B Exemplary image of western blot analysis of HuR protein in NHEM Mock, NHEM BrafV600E and NHEM BrafV600E/HuR OE cells (left). Densitometric quantification of HuR protein in NHEM Mock, NHEM BrafV600E and NHEM BrafV600E/HuR OE cells (Mock = 1) (right). C Exemplary images of H3K9-trimethyl immunofluorescence staining of NHEM Mock, NHEM BrafV600E and NHEM BrafV600E/HuR OE cells (red) and DAPI (blue) staining. CopGFP served as a transduction control (green) (upper). The graph shows the amount of H3K9-trimethyl protein (Mock = 1) (lower). For better recognition, colors are only shown in overlay, not in single pictures. C The graph shows the cell count of NHEM Mock, NHEM BrafV600E and NHEM BrafV600E/HuR OE cells one week after transduction (Mock = 1), Figure S5. The uncropped blots and molecular weight markers of Figure 1D, Figure S6: The uncropped blots and molecular weight markers of Figure 2D, Figure S7: The uncropped blots and molecular weight markers of Figure 3A, Figure S8: The uncropped blots and molecular weight markers of Figure 4E, Figure S9: The uncropped blots and molecular weight markers of Figure 5C, Figure S10: The uncropped blots and molecular weight markers of Figure 7D. Table S1: 150 randomly chosen ARE-containing mRNAs, Table S2: List of primary antibodies, Table S3: List of secondary antibodies.

## References

[1] Siegel R.L., Miller K.D., Jemal A. (2019). Cancer statistics. CA Cancer J. Clin..

[2] Alexandrov L.B., Nik-Zainal S., Wedge D.C., Aparicio S.A., Behjati S., Biankin A.V., Bignell G.R., Bolli N., Borg A., Borresen-Dale A.L. (2013). Signatures of mutational processes in human cancer. Nature.

[3] Bernatchez C., Cooper Z.A., Wargo J.A., Hwu P., Lizee G. (2016). Novel Treatments in Development for Melanoma. Cancer Treat. Res..

[4] Davies H., Bignell G.R., Cox C., Stephens P., Edkins S., Clegg S., Teague J., Woffendin H., Garnett M.J., Bottomley W. (2002). Mutations of the BRAF gene in human cancer. Nature.

[5] Long G.V., Menzies A.M., Nagrial A.M., Haydu L.E., Hamilton A.L., Mann G.J., Hughes T.M., Thompson J.F., Scolyer R.A., Kefford R.F. (2011). Prognostic and clinicopathologic associations of oncogenic BRAF in metastatic melanoma. J. Clin. Oncol..

[6] Pollock P.M., Harper U.L., Hansen K.S., Yudt L.M., Stark M., Robbins C.M., Moses T.Y., Hostetter G., Wagner U., Kakareka J. (2003). High frequency of BRAF mutations in nevi. Nat. Genet..

[7] Abildgaard C., Guldberg P. (2015). Molecular drivers of cellular metabolic reprogramming in melanoma. Trends Mol. Med..

[8] Michaloglou C., Vredeveld L.C., Soengas M.S., Denoyelle C., Kuilman T., van der Horst C.M., Majoor D.M., Shay J.W., Mooi W.J., Peeper D.S. (2005). BRAFE600-associated senescence-like cell cycle arrest of human naevi. Nature.

[9] Kuilman T., Michaloglou C., Mooi W.J., Peeper D.S. (2010). The essence of senescence. Genes Dev..

[10] Narita M., Nunez S., Heard E., Narita M., Lin A.W., Hearn S.A., Spector D.L., Hannon G.J., Lowe S.W. (2003). Rb-mediated heterochromatin formation and silencing of E2F target genes during cellular senescence. Cell.

[11] Fabian M.R., Sonenberg N., Filipowicz W. (2010). Regulation of mRNA translation and stability by microRNAs. Annu. Rev. Biochem..

[12] Eberhardt W., Doller A., Akool E.S., Pfeilschifter J. (2007). Modulation of mRNA stability as a novel therapeutic approach. Pharmacol. Ther..

[13] Ivanov P., Anderson P. (2013). Post-transcriptional regulatory networks in immunity. Immunol. Rev..

[14] Deschenes-Furry J., Angus L.M., Belanger G., Mwanjewe J., Jasmin B.J. (2005). Role of ELAV-like RNA-binding proteins HuD and HuR in the post-transcriptional regulation of acetylcholinesterase in neurons and skeletal muscle cells. Chem. Biol. Interact..

[15] Heinonen M., Bono P., Narko K., Chang S.H., Lundin J., Joensuu H., Furneaux H., Hla T., Haglund C., Ristimaki A. (2005). Cytoplasmic HuR expression is a prognostic factor in invasive ductal breast carcinoma. Cancer Res..

[16] Lopez de Silanes I., Fan J., Yang X., Zonderman A.B., Potapova O., Pizer E.S., Gorospe M. (2003). Role of the RNA-binding protein HuR in colon carcinogenesis. Oncogene.

[17] Blaxall B.C., Dwyer-Nield L.D., Bauer A.K., Bohlmeyer T.J., Malkinson A.M., Port J.D. (2000). Differential expression and localization of the mRNA binding proteins, AU-rich element mRNA binding protein (AUF1) and Hu antigen R (HuR), in neoplastic lung tissue. Mol. Carcinog..

[18] Liaudet N., Fernandez M., Fontao L., Kaya G., Merat R. (2018). Hu antigen R (HuR) heterogeneous expression quantification as a prognostic marker of melanoma. J. Cutan. Pathol..

[19] Dietrich P., Kuphal S., Spruss T., Hellerbrand C., Bosserhoff A.K. (2018). MicroRNA-622 is a novel mediator of tumorigenicity in melanoma by targeting Kirsten rat sarcoma. Pigment. Cell Melanoma Res..

[20] Xu F., Zhang X., Lei Y., Liu X., Liu Z., Tong T., Wang W. (2010). Loss of repression of HuR translation by miR-16 may be responsible for the elevation of HuR in human breast carcinoma. J. Cell Biochem..

[21] Abdelmohsen K., Kim M.M., Srikantan S., Mercken E.M., Brennan S.E., Wilson G.M., Cabo R., Gorospe M. (2010). miR-519 suppresses tumor growth by reducing HuR levels. Cell Cycle.

[22] Guo X., Wu Y., Hartley R.S. (2009). MicroRNA-125a represses cell growth by targeting HuR in breast cancer. RNA Biol..

[23] Al-Ahmadi W., Al-Ghamdi M., Al-Souhibani N., Khabar K.S. (2013). miR-29a inhibition normalizes HuR over-expression and aberrant AU-rich mRNA stability in invasive cancer. J. Pathol..

[24] Petrova N.V., Velichko A.K., Razin S.V., Kantidze O.L. (2016). Small molecule compounds that induce cellular senescence. Aging Cell.

[25] Haferkamp S., Borst A., Adam C., Becker T.M., Motschenbacher S., Windhovel S., Hufnagel A.L., Houben R., Meierjohann S. (2013). Vemurafenib induces senescence features in melanoma cells. J. Investig. Dermatol..

[26] Bernardi R., Pandolfi P.P. (2007). Structure, dynamics and functions of promyelocytic leukaemia nuclear bodies. Nat. Rev. Mol. Cell Biol.

[27] Giacinti C., Giordano A. (2006). RB and cell cycle progression. Oncogene.

[28] Kunz M., Loffler-Wirth H., Dannemann M., Willscher E., Doose G., Kelso J., Kottek T., Nickel B., Hopp L., Landsberg J. (2018). RNA-seq analysis identifies different transcriptomic types and developmental trajectories of primary melanomas. Oncogene.

[29] Wurth L. (2012). Versatility of RNA-Binding Proteins in Cancer. Comp. Funct. Genom..

[30] Moore M.J. (2005). From birth to death: The complex lives of eukaryotic mRNAs. Science.

[31] Kim M.Y., Hur J., Jeong S. (2009). Emerging roles of RNA and RNA-binding protein network in cancer cells. BMB Rep..

[32] Silvera D., Formenti S.C., Schneider R.J. (2010). Translational control in cancer. Nat. Rev. Cancer.

[33] Moradi F., Berglund P., Linnskog R., Leandersson K., Andersson T., Prasad C.P. (2016). Dual mechanisms of action of the RNA-binding protein human antigen R explains its regulatory effect on melanoma cell migration. Transl. Res..

[34] Hatanaka T., Higashino F., Tei K., Yasuda M. (2019). The neural ELAVL protein HuB enhances endogenous proto-oncogene activation. Biochem. Biophys. Res. Commun..

[35] Battaglia-Hsu S.F., Ghemrawi R., Coelho D., Dreumont N., Mosca P., Hergalant S., Gauchotte G., Sequeira J.M., Ndiongue M., Houlgatte R. (2018). Inherited disorders of cobalamin metabolism disrupt nucleocytoplasmic transport of mRNA through impaired methylation/phosphorylation of ELAVL1/HuR. Nucleic Acids Res..

[36] Zhou X., Wang S., Zheng M., Kuver A., Wan X., Dai K., Li X. (2019). Phosphorylation of ELAVL1 (Ser219/Ser316) mediated by PKC is required for erythropoiesis. Biochim. Biophys. Acta Mol. Cell Res..

[37] Denkert C., Weichert W., Winzer K.J., Muller B.M., Noske A., Niesporek S., Kristiansen G., Guski H., Dietel M., Hauptmann S. (2004). Expression of the ELAV-like protein HuR is associated with higher tumor grade and increased cyclooxygenase-2 expression in human breast carcinoma. Clin. Cancer Res..

[38] Sohn B.H., Park I.Y., Lee J.J., Yang S.J., Jang Y.J., Park K.C., Kim D.J., Lee D.C., Sohn H.A., Kim T.W. (2010). Functional switching of TGF-beta1 signaling in liver cancer via epigenetic modulation of a single CpG site in TTP promoter. Gastroenterology.

[39] Abdelmohsen K., Srikantan S., Kuwano Y., Gorospe M. (2008). miR-519 reduces cell proliferation by lowering RNA-binding protein HuR levels. Proc. Natl. Acad. Sci. USA.

[40] Stark M.S., Tyagi S., Nancarrow D.J., Boyle G.M., Cook A.L., Whiteman D.C., Parsons P.G., Schmidt C., Sturm R.A., Hayward N.K. (2010). Characterization of the Melanoma miRNAome by Deep Sequencing. PLoS ONE.

[41] Poell J.B., van Haastert R.J., de Gunst T., Schultz I.J., Gommans W.M., Verheul M., Cerisoli F., van Puijenbroek A., van Noort P.I., Prevost G.P. (2013). Correction: A Functional Screen Identifies Specific MicroRNAs Capable of Inhibiting Human Melanoma Cell Viability. PLoS ONE.

[42] Zhang Z., Zhang S., Ma P., Jing Y., Peng H., Gao W.Q., Zhuang G. (2015). Lin28B promotes melanoma growth by mediating a microRNA regulatory circuit. Carcinogenesis.

[43] Serini S., Fasano E., Piccioni E., Monego G., Cittadini A.R., Celleno L., Ranelletti F.O., Calviello G. (2012). DHA induces apoptosis and differentiation in human melanoma cells in vitro: Involvement of HuR-mediated COX-2 mRNA stabilization and beta-catenin nuclear translocation. Carcinogenesis.

[44] Malumbres M., Barbacid M. (2009). Cell cycle, CDKs and cancer: A changing paradigm. Nat. Rev. Cancer.

[45] Wang W., Yang X., Cristofalo V.J., Holbrook N.J., Gorospe M. (2001). Loss of HuR is linked to reduced expression of proliferative genes during replicative senescence. Mol. Cell Biol..

[46] Lal A., Mazan-Mamczarz K., Kawai T., Yang X., Martindale J.L., Gorospe M. (2004). Concurrent versus individual binding of HuR and AUF1 to common labile target mRNAs. EMBO J..

[47] Del Vecchio G., De Vito F., Saunders S.J., Risi A., Mannironi C., Bozzoni I., Presutti C. (2016). RNA-binding protein HuR and the members of the miR-200 family play an unconventional role in the regulation of c-Jun mRNA. RNA.

[48] Merat R., Bugi-Marteyn A., Wrobel L.J., Py C., Daali Y., Schwarzler C., Liaudet N. (2019). Drug-induced expression of the RNA-binding protein HuR attenuates the adaptive response to BRAF inhibition in melanoma. Biochem. Biophys. Res. Commun..

[49] Lee J.H., Jung M., Hong J., Kim M.K., Chung I.K. (2018). Loss of RNA-binding protein HuR facilitates cellular senescence through posttranscriptional regulation of TIN2 mRNA. Nucleic Acids Res..

[50] Hoek K.S., Eichhoff O.M., Schlegel N.C., Dobbeling U., Kobert N., Schaerer L., Hemmi S., Dummer R. (2008). In vivo switching of human melanoma cells between proliferative and invasive states. Cancer Res..

[51] Bertolotto C., Lesueur F., Giuliano S., Strub T., de Lichy M., Bille K., Dessen P., d’Hayer B., Mohamdi H., Remenieras A. (2011). A SUMOylation-defective MITF germline mutation predisposes to melanoma and renal carcinoma. Nature.

[52] Hartman M.L., Czyz M. (2015). MITF in melanoma: Mechanisms behind its expression and activity. Cell Mol. Life Sci..

[53] Gray-Schopfer V.C., Cheong S.C., Chong H., Chow J., Moss T., Abdel-Malek Z.A., Marais R., Wynford-Thomas D., Bennett D.C. (2006). Cellular senescence in naevi and immortalisation in melanoma: A role for p16?. Br. J. Cancer.

[54] Kim G., Meriin A.B., Gabai V.L., Christians E., Benjamin I., Wilson A., Wolozin B., Sherman M.Y. (2012). The heat shock transcription factor Hsf1 is downregulated in DNA damage-associated senescence, contributing to the maintenance of senescence phenotype. Aging Cell.

[55] Meisner N.C., Hintersteiner M., Mueller K., Bauer R., Seifert J.M., Naegeli H.U., Ottl J., Oberer L., Guenat C., Moss S. (2007). Identification and mechanistic characterization of low-molecular-weight inhibitors for HuR. Nat. Chem. Biol..

[56] Wu X., Lan L., Wilson D.M., Marquez R.T., Tsao W.C., Gao P., Roy A., Turner B.A., McDonald P., Tunge J.A. (2015). Identification and validation of novel small molecule disruptors of HuR-mRNA interaction. ACS Chem. Biol..

[57] Blanco F.F., Preet R., Aguado A., Vishwakarma V., Stevens L.E., Vyas A., Padhye S., Xu L., Weir S.J., Anant S. (2016). Impact of HuR inhibition by the small molecule MS-444 on colorectal cancer cell tumorigenesis. Oncotarget.

[58] Lang M., Berry D., Passecker K., Mesteri I., Bhuju S., Ebner F., Sedlyarov V., Evstatiev R., Dammann K., Loy A. (2017). HuR Small-Molecule Inhibitor Elicits Differential Effects in Adenomatosis Polyposis and Colorectal Carcinogenesis. Cancer Res..

[59] Braig S., Mueller D.W., Bosserhoff A.K. (2010). Micro RNA miR-196a is a central regulator of HOX-B7 and BMP4 expression in malignant melanoma. Cell. Mol. Life Sci..

[60] Feuerer L., Lamm S., Henz I., Kappelmann-Fenzl M., Haferkamp S., Meierjohann S., Hellerbrand C., Kuphal S., Bosserhoff A.K. (2019). Role of melanoma inhibitory activity in melanocyte senescence. Pigment. Cell Melanoma Res..

[61] Ma W., Mayr C. (2018). A Membraneless Organelle Associated with the Endoplasmic Reticulum Enables 3′UTR-Mediated Protein-Protein Interactions. Cell.

[62] Hannus M., Beitzinger M., Engelmann J.C., Weickert M.T., Spang R., Hannus S., Meister G. (2014). siPools: Highly complex but accurately defined siRNA pools eliminate off-target effects. Nucleic Acids Res..

[63] Schiffner S., Braunger B.M., de Jel M.M., Coupland S.E., Tamm E.R., Bosserhoff A.K. (2014). Tg(Grm1) transgenic mice: A murine model that mimics spontaneous uveal melanoma in humans?. Exp. Eye Res..

[64] Dietrich P., Kuphal S., Spruss T., Hellerbrand C., Bosserhoff A.K. (2018). Wild-type KRAS is a novel therapeutic target for melanoma contributing to primary and acquired resistance to BRAF inhibition. Oncogene.

[65] Ott C.A., Linck L., Kremmer E., Meister G., Bosserhoff A.K. (2016). Induction of exportin-5 expression during melanoma development supports the cellular behavior of human malignant melanoma cells. Oncotarget.

[66] Linck L., Liebig J., Voller D., Eichner N., Lehmann G., Meister G., Bosserhoff A. (2018). MicroRNA-sequencing data analyzing melanoma development and progression. Exp. Mol. Pathol..

[67] Bohme I., Bosserhoff A. (2020). Extracellular acidosis triggers a senescence-like phenotype in human melanoma cells. Pigment. Cell Melanoma Res..

[68] Ruedel A., Dietrich P., Schubert T., Hofmeister S., Hellerbrand C., Bosserhoff A.K. (2015). Expression and function of microRNA-188-5p in activated rheumatoid arthritis synovial fibroblasts. Int. J. Clin. Exp. Pathol..

[69] Arndt S., Wacker E., Li Y.F., Shimizu T., Thomas H.M., Morfill G.E., Karrer S., Zimmermann J.L., Bosserhoff A.K. (2013). Cold atmospheric plasma, a new strategy to induce senescence in melanoma cells. Exp. Dermatol..

[70] Kappelmann M., Kuphal S., Meister G., Vardimon L., Bosserhoff A.K. (2013). MicroRNA miR-125b controls melanoma progression by direct regulation of c-Jun protein expression. Oncogene.

[71] Franken N.A., Rodermond H.M., Stap J., Haveman J., van Bree C. (2006). Clonogenic assay of cells in vitro. Nat. Protoc..

[72] Roff A.N., Craig T.J., August A., Stellato C., Ishmael F.T. (2014). MicroRNA-570-3p regulates HuR and cytokine expression in airway epithelial cells. Am. J. Clin. Exp. Immunol..

[73] Anaya J. (2016). OncoLnc: Linking TCGA survival data to mRNAs, miRNAs, and lncRNAs. PeerJ Comput. Sci..

