# Supplementary material for: HuRdling Senescence: HuR Breaks BRAF-Induced Senescence in Melanocytes and Supports Melanoma Growth
Source: Cancers (Basel). 2020 May 21;12(5):1299. doi: 10.3390/cancers12051299 (PMC7281285; doi:10.3390/cancers12051299)
Supplement: Supplementary file 1 [file cancers-12-01299-s001.pdf]

Supplementary Materials

HuRdling Senescence: HuR Breaks BRAF-Induced Senescence in Melanocytes and Supports Melanoma Growth

Janika K. Liebig, Silke Kuphal and Anja Katrin Bosserhoff

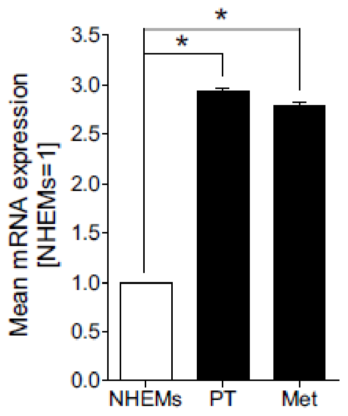

Figure S1. Mean expression of mRNAs in NHEMs and primary and metastatic melanoma cells. Data from cDNA array (Gene Omnibus (GEO) database (GSE108969)). Expression in NHEMs = 1. Expression levels of 28536 genes were involved in the calculation.

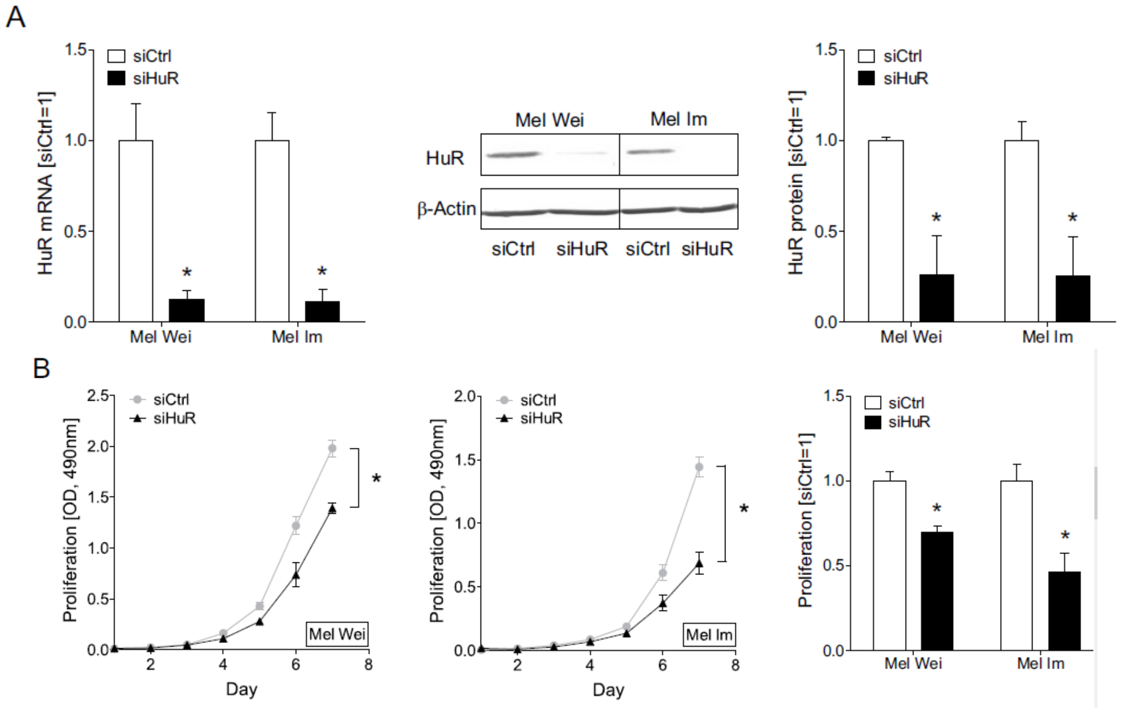

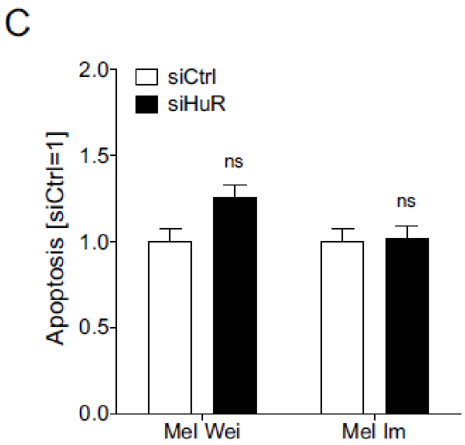

Figure S2. A Transfection control for siHuRpool and siCtrlpool transfection. Relative expression of HuR mRNA in transfected Mel Wei and Mel Im cells (siCtrl=1) (left). Densitometric quantification (right) and exemplary image (middle) of western blot analysis of HuR protein levels in transfected Mel Wei and Mel Im cells (siCtrl=1). **B** Exemplary proliferation curves of Mel Wei and Mel Im cells resulting from a XTT assay (left panel) and quantified 'OD' (proliferative ability, siCtrl=1) (right panel). **C** FACS-based analysis of apoptosis in transfected Mel Wei and Mel Im cells (siCtrl=1).

Transfection control for siHuRpool and siCtrlpool transfection (shown membrane=> n=1)

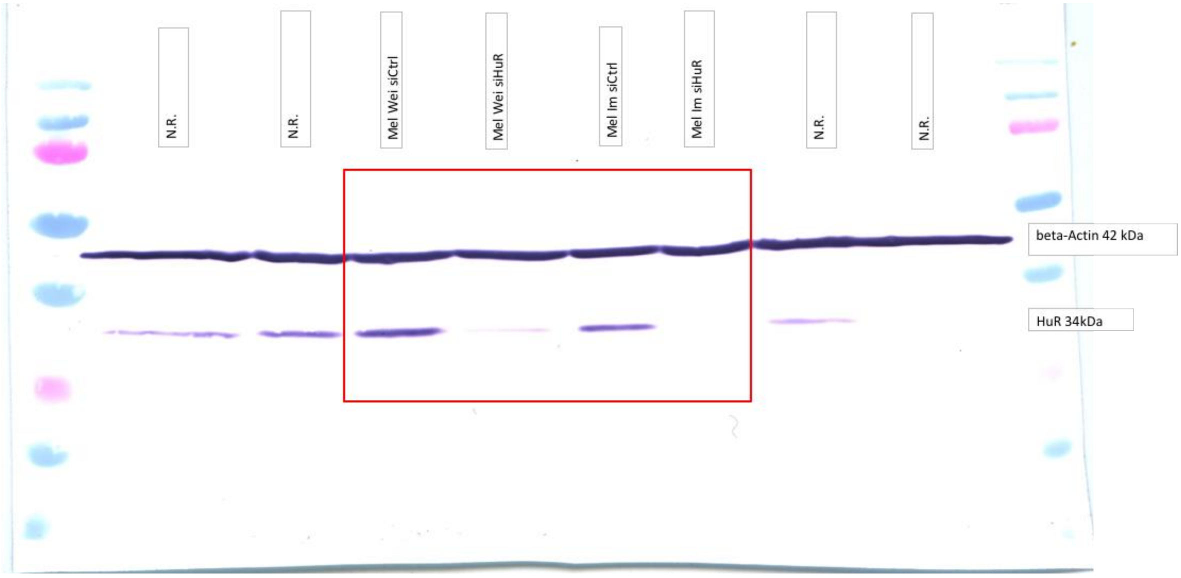

HuR normal. to beta-Actin:

	n=1	n=2	n=3
Mel Wei siCtrl	1.934.363	1.715.308	1.646.097
Mel Wei siHuR	345.849	714.881	568.587
Mel Im siCtrl	6.601.167	1.563.972	1.924.312
Mel Im siHuR	77.536	547.075	761.333

The uncropped blots and molecular weight markers of Figure S2A. (Samples labeled with “N.R.“ (not relevant) are part of other studies and not relevant for this study)

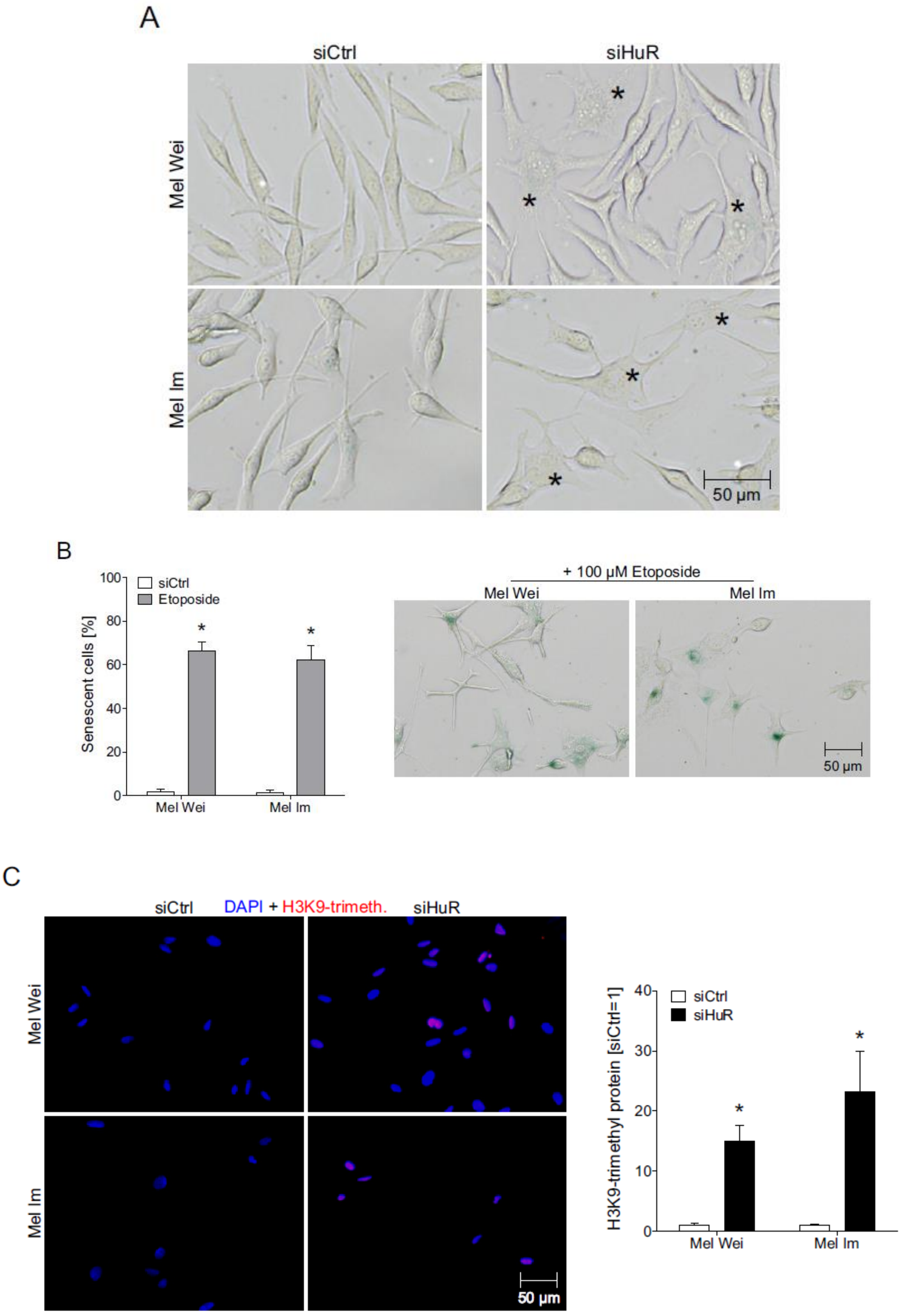

Figure S3. A Exemplary images of light microscopic examination of siCtrl and siHuR transfected Mel Wei and Mel Im cells. Enlarged cells marked with asterisks. **B** Exemplary images of light microscopic examination of SA-β-galactosidase staining in etoposide treated Mel Wei and Mel Im cells (positive control) (right). The percentages of SA-β-galactosidase positive cells (blue) were calculated (left) (siCtrl=1). **C** Exemplary images of H3K9-trimethyl immunofluorescence staining of Mel Wei and Mel Im cells. Panels show overlays of H3K9-trimethyl (red) and DAPI (blue) staining (left). The graph shows the amount of H3K9-trimethyl protein (right) (siCtrl=1).

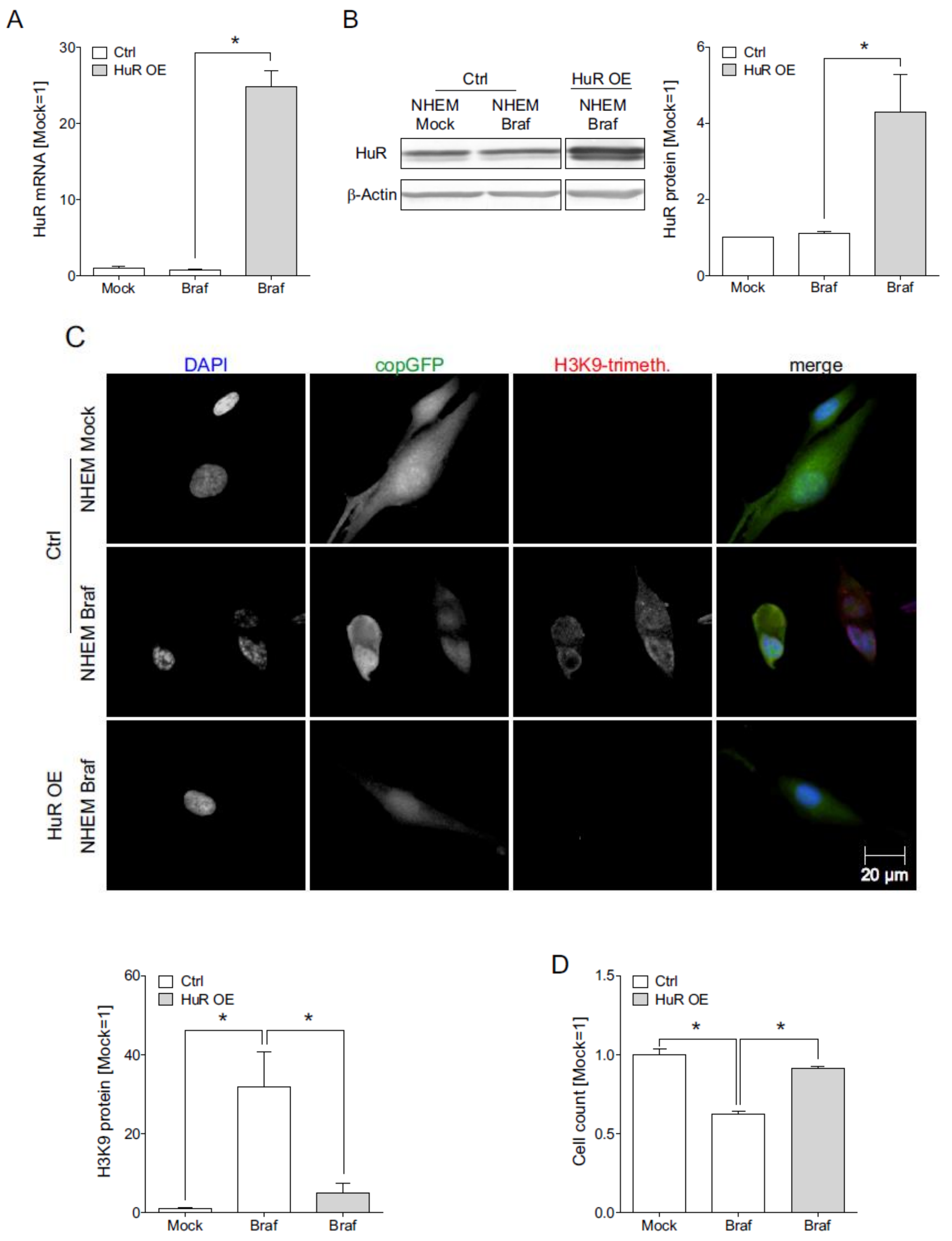

Figure S4. A Transduction control for HuR OE. Relative expression of HuR mRNA in NHEM Mock, NHEM *Braf*^{V600E} and NHEM *Braf*^{V600E}/HuR OE cells (Mock=1). **B** Exemplary image of western blot analysis of HuR protein in NHEM Mock, NHEM *Braf*^{V600E} and NHEM *Braf*^{V600E}/HuR OE cells (left). Densitometric quantification of HuR protein in NHEM Mock, NHEM *Braf*^{V600E} and NHEM *Braf*^{V600E}/HuR OE cells (Mock=1) (right). **C** Exemplary images of H3K9-trimethyl immunofluorescence staining of NHEM Mock, NHEM *Braf*^{V600E} and NHEM *Braf*^{V600E}/HuR OE cells (red) and DAPI (blue) staining. CopGFP served as a transduction control (green) (upper). The graph shows the amount of H3K9-trimethyl protein (Mock=1) (lower). For better recognition, colors are only shown in overlay, not in single pictures. **C** The graph shows the cell count of NHEM Mock, NHEM *Braf*^{V600E} and NHEM *Braf*^{V600E}/HuR OE cells one week after transduction (Mock=1).

Transduction control for lentiviral HuR Overexpression (shown membrane=> n=1)

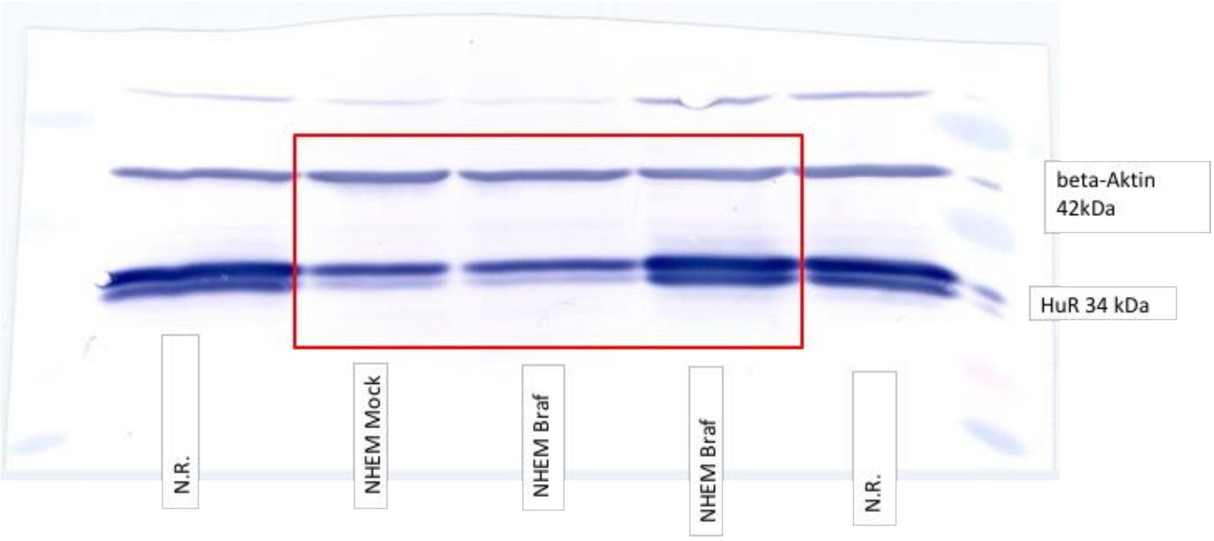

HuR normal. to beta-Actin:

	n=1	n=2	n=3
Mock	73.723	68.079	251.064
Braf	90.413	90.865	301.502
Braf HuR	368.871	379.654	583.322

The uncropped blots and molecular weight markers of Figure S4B. (Samples labeled with “N.R.“ (not relevant) are part of other studies and not relevant for this study)

HuR level in melanoma cells compared to NHEMs (shown membrane=> n=1)

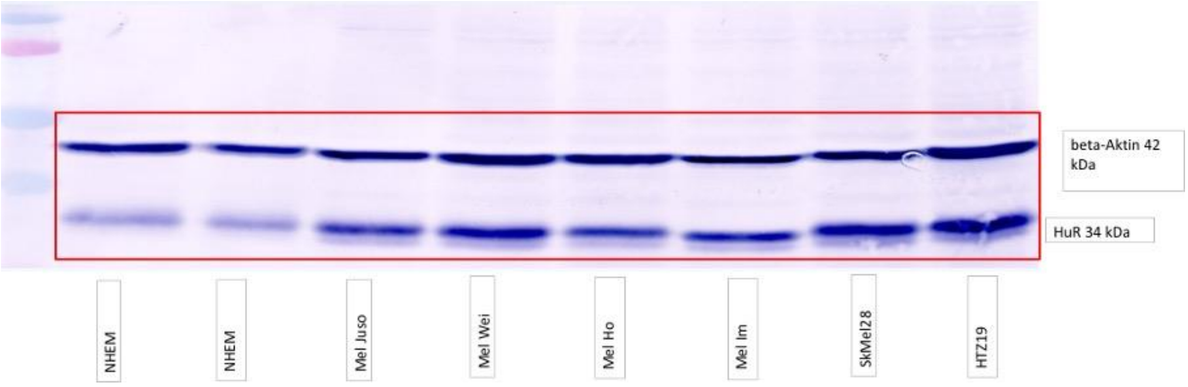

beta-Actin and HuR:

⇨ n=1:

	beta-Aktin	HuR
NHEM p7	5.491.527	3.486.933
NHEM p6	5.082.062	3.609.518
Mel Juso	5.597.648	6.992.468
Mel Wei	7.042.770	9.053.468
Mel Ho	6.399.820	7.062.640
Mel Im	5.366.113	6.655.983
SkMel28	6.164.941	10.110.468
HTZ19	8.742.376	12.224.125

⇨ n=2:

	beta-Aktin	HuR
NHEM p7	5.864.891	1.573.134
NHEM p7	6.608.770	3.973.740
NHEM p7	7.938.305	4.924.397
Mel Wei	7.274.355	8.249.205
Mel Ho	6.681.426	5.425.225
Mel Im	5.836.648	5.240.205
SkMel28	5.335.991	6.175.497
HTZ19	6.682.527	5.189.205

⇨ n=3:

	HuR	beta-Aktin
NHEM p7	23.223.966	70.051.693
NHEM p8	53.791.087	98.531.208
Mel Juso	59.350.673	86.227.844
Mel Wei	73.872.329	106.511.865
Mel Ho	43.851.702	79.388.995
Mel Im	60.087.551	32.745.066
SkMel28	64.926.421	75.291.208
Htz19d	70.972.421	74.184.300

Figure S5. The uncropped blots and molecular weight markers of Figure 1D. (Samples labeled with "N.R." (not relevant) are part of other studies and not relevant for this study).

HuR level in melanoma cells after miR-194-mimic transfection (shown membranes=> n=1)

For this blot, ECL western blot detection was used. The western blot marker is cropped into the membrane via the ECL software during detection of the bands.

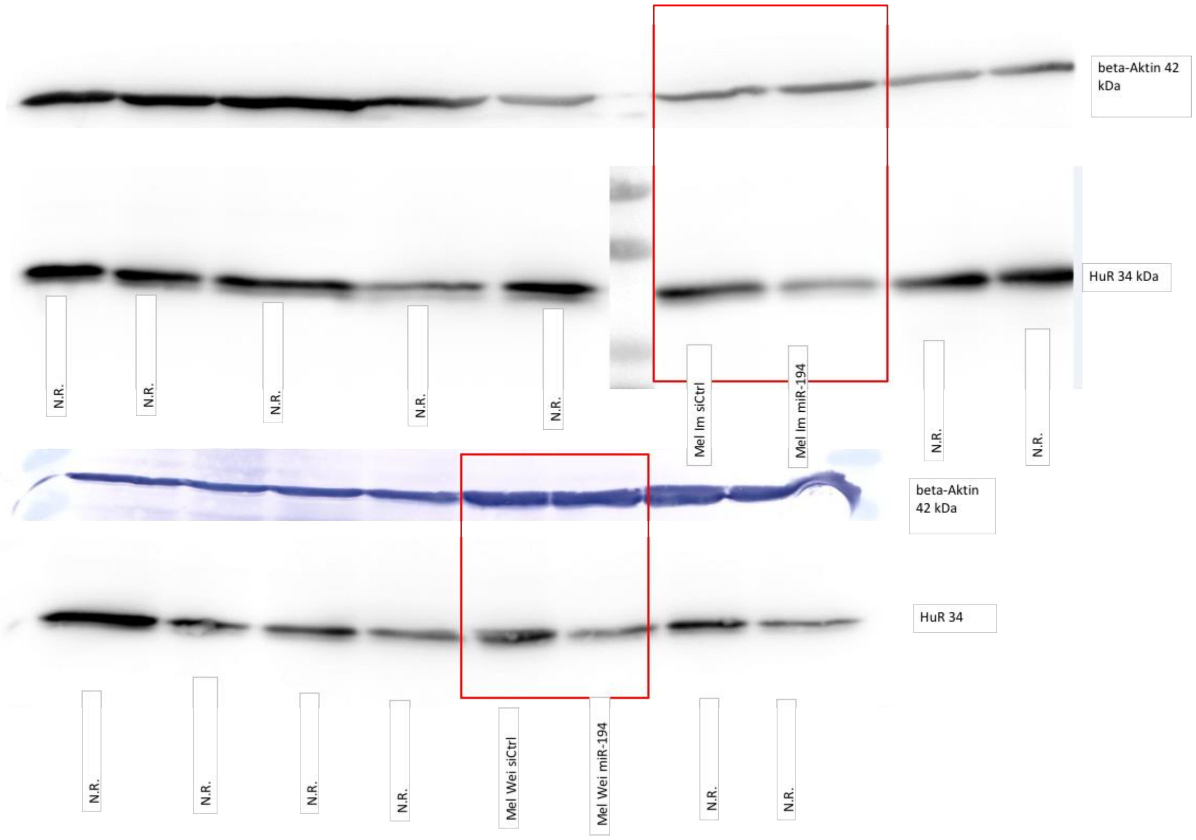

beta-Actin and HuR:

1		HuR	beta-Act
	Mel Wei siCtrl	1.591.956	139368816
	Mel Wei miR-194	1.561.157	213711616
	Mel Im siCtrl	505.117	40542540
	Mel Im miR-194	664.387	97301816
2		HuR	beta-Act
	Mel Wei siCtrl	323.045.792	191.124.736
	Mel Wei miR-194	148.046.912	199.514.192
	Mel Im siCtrl	334.201.824	129.838.936
	Mel Im miR-194	303.958.048	177.035.024
3		HuR	beta-Act
	Mel Wei siCtrl	137.562.688	65.166.408
	Mel Wei miR-194	82.440.512	45.094.860
	Mel Im siCtrl	68.585.432	4.829.851
	Mel Im miR-194	82.180.880	19.273.710

Figure S6. The uncropped blots and molecular weight markers of Figure 2D. (Samples labeled with "N.R." (not relevant) are part of other studies and not relevant for this study).

HuR localization in melanoma cells and NHEM (Nucleus/Cytoplasm) (shown membranes=> n=1)
No quantification!

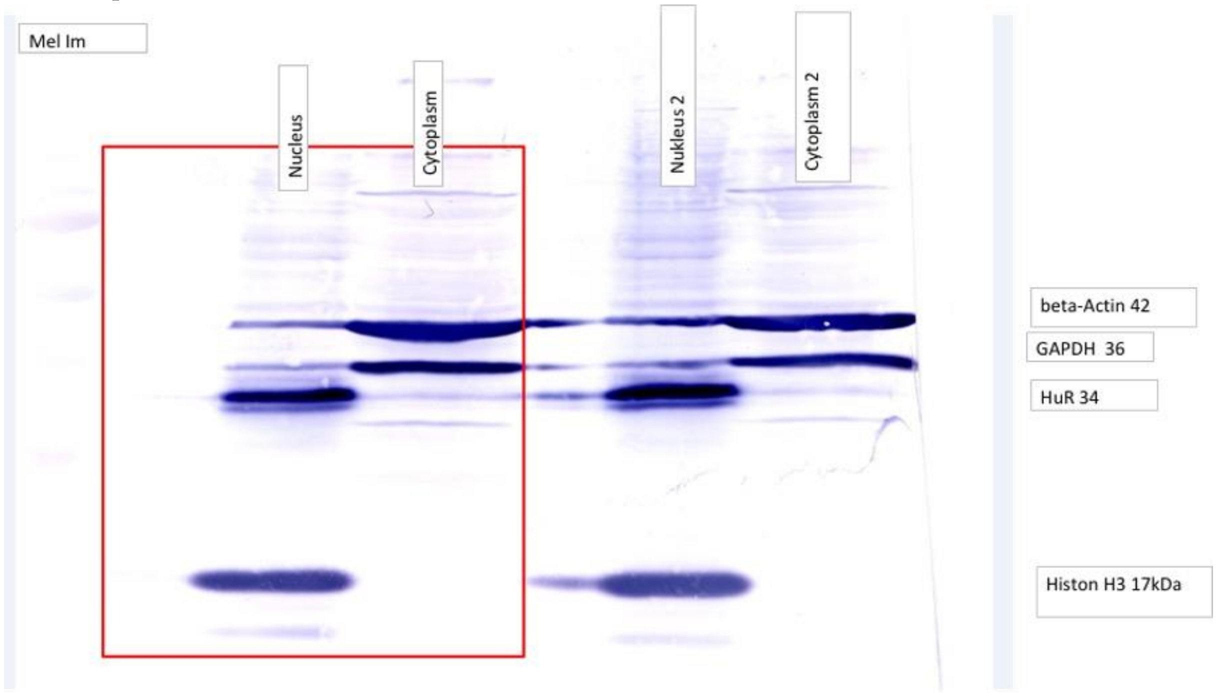

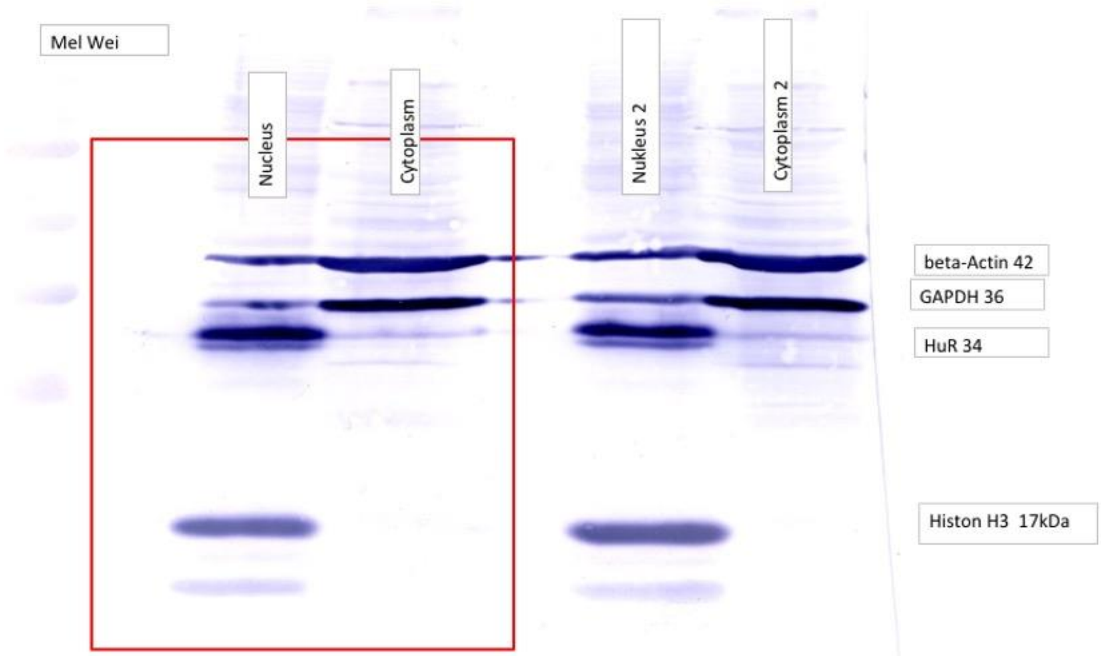

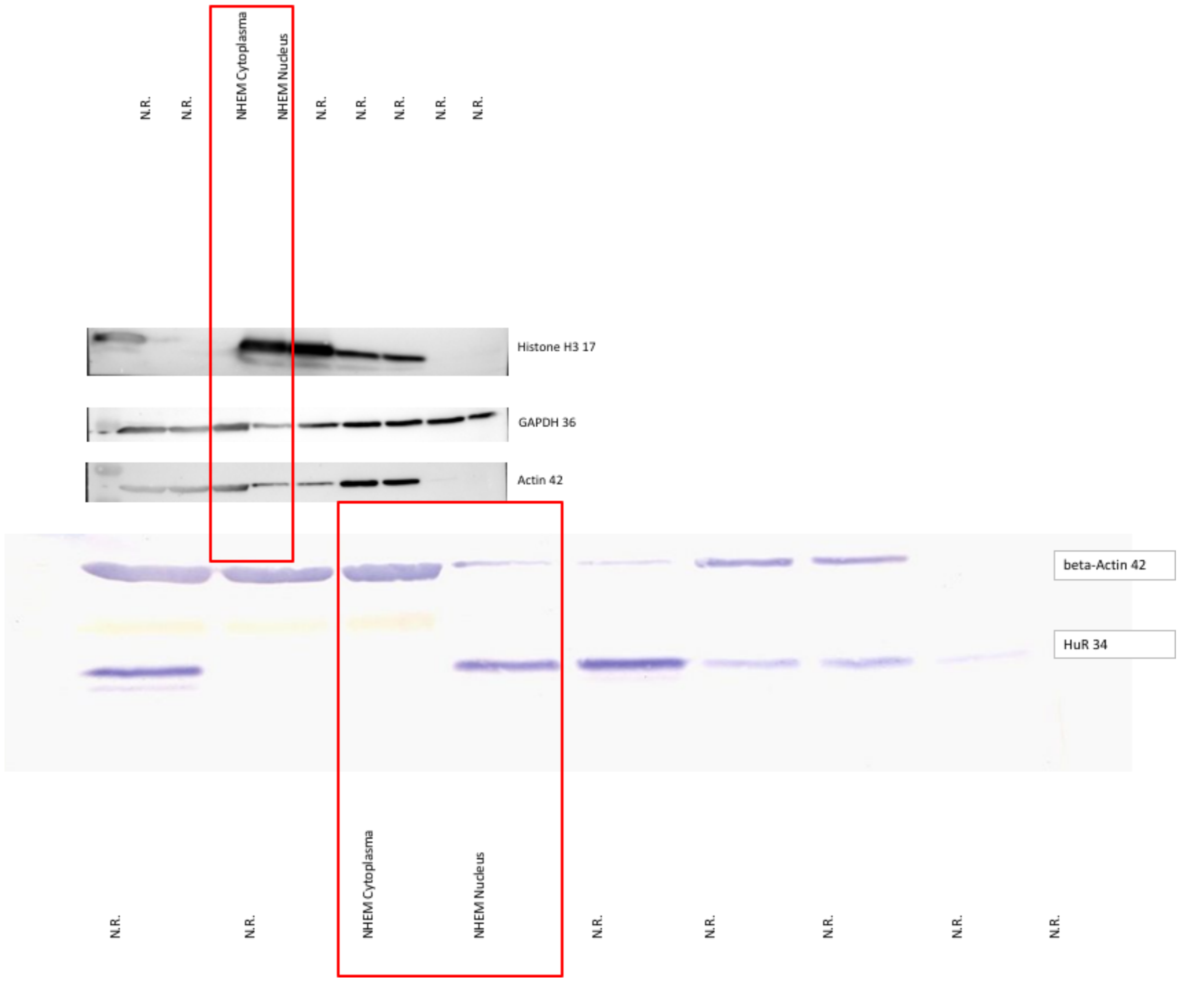

Figure S7. The uncropped blots and molecular weight markers of Figure 3A. (Samples labeled with "N.R." (not relevant) are part of other studies and not relevant for this study).

Influence of siHuRpool KD on cyclin D1 level in melanoma cells (shown membranes=> n=1)

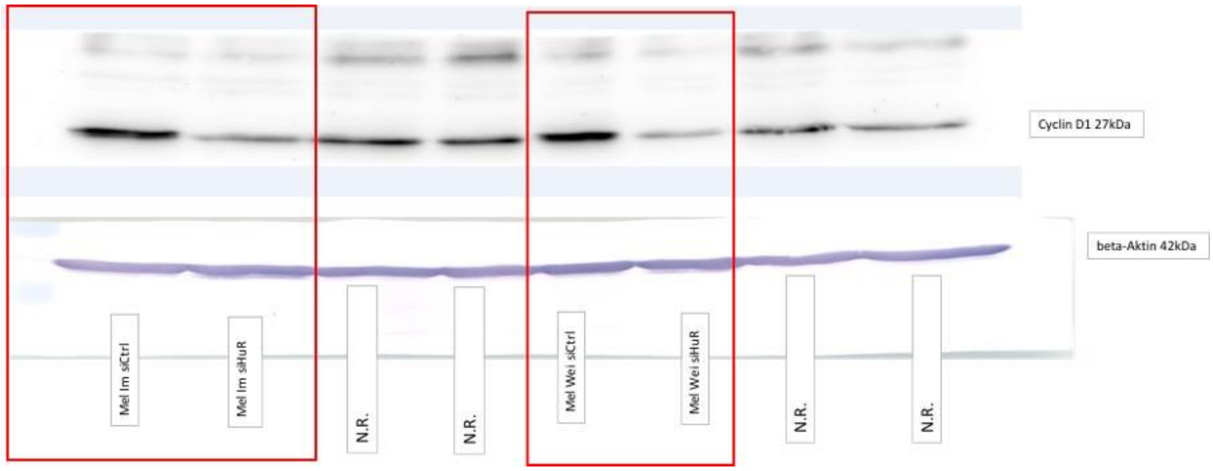

CyclinD1 normal. to beta-Actin:

	siCtrl						siHuR					
Mel Wei	1092751	694782	2409110	2009810	1020877	757339	544379	383328	1134012	624239	388652	528407
Mel Im	1301718	949003	1996500	1327007	1610510	803924	423258	445885	1557270	640211	918390	644204

Figure S8. The uncropped blots and molecular weight markers of Figure 4E. (Samples labeled with "N.R." (not relevant) are part of other studies and not relevant for this study).

Influence of siHuRpool KD on MITF level in melanoma cells (shown membranes=> n=1)

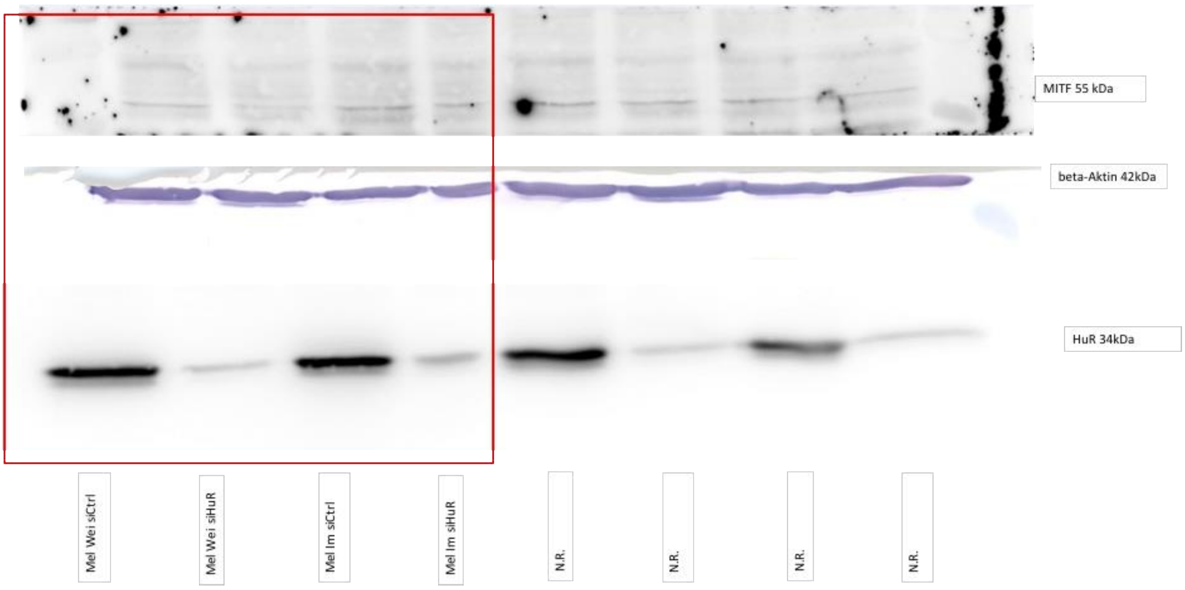

MITF normal. to beta-Actin:

	Volumen
Mel Im siCtrl	480.829
Mel Im siHuR	308.088
Mel Wei siCtrl	229.848
Mel Wei siHuR	122.196
Mel Im siCtrl	1.032.464
Mel Im siHuR	676.200
Mel Wei siCtrl	1.553.024
Mel Wei siHuR	813.741

Figure S9. The uncropped blots and molecular weight markers of Figure 5C. (Samples labeled with “N.R.“ (not relevant) are part of other studies and not relevant for this study).

Influence of lentiviral HuR overexpression on MITF level in NHEMs (shown membranes=> n=1) (see also Figure S4 for HuR & beta-Act)

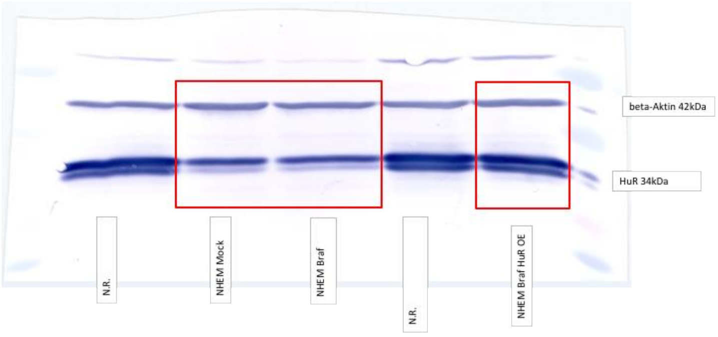

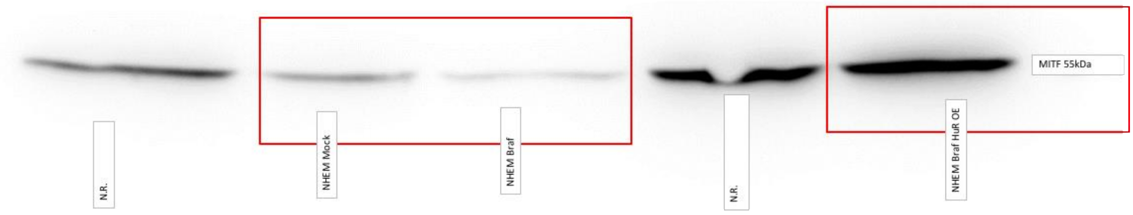

MITF normal. to beta-Actin:

NHEM Mock	1189,406	926,0586	2188,756
NHEM BRAF	1430,871	280,1593	2205,545
NHEM BRAF HuR OE	379,7224	179,3351	2441,641

Figure S10. The uncropped blots and molecular weight markers of Figure 7D. (Samples labeled with "N.R." (not relevant) are part of other studies and not relevant for this study).

Table S1. 150 randomly chosen ARE-containing mRNAs.

ELOVL5	E2F7	CALCOCO1
NRAS	ADAT1	EHD2
CYP51A1	WAPAL	IFT88
ZZZ3	MAP2K1	EPHA3
ZFYVE16	DERA	YAF2
PTBP1	LAP3	ZNF200
CCND1	ADSS	NOTCH3
ABCC2	CCDC90A	PPP1R3F
SLAMF7	MAPKAP1	MAPK4
RGPD5	CCND3	SMAD6
PTEN	NUP160	CRAMP1L
RANBP9	C1orf124	MAP9
INSIG1	MAP4K4	MAP3K13
DICER1	RABGAP1	RGNEF
NOTCH2NL	FOXN3	MAP7
MAPRE1	GCLM	TIMP2
IBTK	PLAUR	MYO16
ZC3H15	TP53RK	IL6
CREB1	GABRA3	PKP2
TNPO3	FAM120A	MAP2K6
CUL3	RALBP1	ITGB2
MAPK6	ITGA4	PRDM1
KPNA6	ARID1B	IL13
KIAA0776	FAM76A	BCAS1
MAP3K7	ITGA2	COL11A1
ATP11B	ETV1	CCDC85A
PKN2	HSF2	SLC18A1
SKAP2	KIAA1539	CDKL3
HEATR5B	PER3	XK
CCDC99	AIFM2	IL10
PHTF2	PRKAR2B	MAP3K15
TRAF3IP2	CCDC132	IYD
MBTPS2	CDON	ADAM22
SLC12A2	C2orf3	ODZ1
TRIO	E2F5	ARHGAP6
SLC30A9	FAS	ITGB6
RIOK2	INSIG2	USH2A
MON2	MAP3K4	ASB4
UBR2	CYFIP2	ADAMTS6
DCUN1D1	MAP3K12	LRRC7
EIF2AK2	KIAA1370	PAX6
NSUN2	STAU2	ADAM28
TCEB3	SMAD3	CD38
SLK	ARID4B	UPP2
PRKCH	E2F8	HGF
KIF1B	ARID4A	CDH10
PGM3	ATP9A	LY75
C12orf4	MCM10	TFPI
CYR61	ST7L	IL15
SMAD2	CENPQ	MSH4

Table S2. List of primary antibodies.

Antigen/Size	Species	Method	Dilution	Manufacturer
β-Actin (42)	*mouse*	WB	1:5000 in TBS-T	Sigma-Aldrich Chemie GmbH, Steinheim, Germany
Cyclin D1 (34 - 40)	*mouse*	WB	1:1000 in 5 % MP / TBS-T	Santa Cruz Biotechnology, Inc., Dallas, USA
GAPDH (36)	*rabbit*	WB	1:1000 in 5 % BSA / TBS-T	Cell Signaling Technology, Danvers, MA, USA
H3K9-trimethyl. (17)	*rabbit*	IF	1:200 in PBS	Merck, Darmstadt, Germany
Histon H3 (15)	*rabbit*	WB	1:2000 in 5 % BSA / TBS-T	Abcam, Cambridge, England
HuR (32)	*mouse*	WB/IF/IH	1:4000 in 5 % BSA / TBS-T (WB) 1:500 in PBS (IF) 1: 50 (IH)	Santa Cruz Biotechnology, Inc., Dallas, USA
Ki-67 (320)	*mouse*	IF	1:100 in PBS	BD Biosciences, Heidelberg, Germany
MITF (55)	*mouse*	WB	1:1000 in 5 % BSA / TBS-T	Santa Cruz Biotechnology, Inc., Dallas, USA
PML (78, 97)	*mouse*	IF	1:200 in PBS	Santa Cruz Biotechnology, Inc., Dallas, USA

Table S3. List of secondary antibodies.

Name/Species	Method	Dilution	Manufacturer
Anti-*Mouse*-AP	WB	1:3000 in TBS-T	Cell Signaling Technology, Danvers, MA, USA
Anti-*Mouse*-HRP	WB	1:3000 in TBS-T	Cell Signaling Technology, Danvers, MA, USA
Anti-*Rabbit*-AP	WB	1:3000 in TBS-T	Cell Signaling Technology, Danvers, MA, USA
Anti-*Rabbit*-HRP	WB	1:3000 in TBS-T	Cell Signaling Technology, Danvers, MA, USA
Alexa Fluor Plus 555 (*mouse)*	IF	1:500 in PBS	Thermo Fisher Scientific, Waltham, MA, USA
Alexa Fluor Plus 555 (*rabbit)*	IF	1:1000 in PBS	Thermo Fisher Scientific, Waltham, MA, USA

© 2020 by the authors. Licensee MDPI, Basel, Switzerland. This article is an open access article distributed under the terms and conditions of the Creative Commons Attribution (CC BY) license (http://creativecommons.org/licenses/by/4.0/).
